# Machine learning for predicting mortality in patients with sepsis and its associated complications: A systematic review and meta-analysis

**DOI:** 10.1371/journal.pone.0362057

**Published:** 2026-11-02

**Authors:** Hanxi Lv, Yan Gao, Aiping Yu

**Affiliations:** Department of Respiratory and Critical Care Medicine, West China Hospital, Sichuan University, Chengdu, Sichuan, China; Children’s National Hospital, George Washington University, UNITED STATES OF AMERICA

## Abstract

**Background:**

Sepsis remains a leading cause of death in intensive care units worldwide and may be accompanied by complex complications, including encephalopathy, coagulopathy, acute kidney injury, myocardial dysfunction, and delirium, each of which substantially increases the risk of poor outcomes. Machine learning, a promising approach for prognostic prediction, can capture complex nonlinear relationships among clinical risk factors. However, its predictive performance in sepsis and its specific complications remains unclear.

**Method:**

We systematically searched PubMed, Embase, Web of Science, and the Cochrane Library for relevant literature through April 2026. Studies that aimed to develop or validate machine learning models to predict mortality in adult patients with sepsis and its associated complications were included. The random-effects or bivariate model was used to calculate the AUC, sensitivity, and specificity.

**Results:**

Overall, machine learning models show moderate predictive performance for short-term mortality in sepsis, with a combined AUC of 0.82 for the training set (46 studies, 15 models) and 0.79 for the validation set (50 studies, 25 models). Logistic regression is the most widely evaluated model, demonstrating stable performance (validation AUC = 0.81). Integrated methods, including random forest and XGBoost, performed equally well, with AUCs between 0.81 and 0.83 in both groups. Although LightGBM and DL-CMT achieved the highest AUCs in the training and validation sets, respectively, both estimates were based on a single study.

Among the complications, the evidence for sepsis-associated encephalopathy (AUC = 0.81, 3 studies) and sepsis-induced coagulopathy (AUC = 0.80, 4 studies) is the most conclusive and best demonstrated. Sepsis-associated acute kidney injury showed moderate and stable performance (validation AUC = 0.79, 4 studies). Sepsis-induced cardiomyopathy showed significant attenuation from training to validation (AUC 0.84 vs 0.78, all from a single study), and random forest performed well in training (AUC = 0.98) but was not widely reported. Septic shock reached the highest AUC (0.95), but this was based on a single study. The predictive utility of sepsis-associated delirium is limited (AUC = 0.67, 1 study), and sepsis-induced myocardial injury is moderate (AUC = 0.77, 1 study).For long-term mortality, evidence is extremely lacking. Only SAE (AUC 0.88 during training and 0.86 during validation) and SCRS (combined AUC 0.76) provided single study estimates, ruling out reliable conclusions.

**Conclusion:**

In this meta-analysis, machine learning models showed moderate predictive performance for short-term mortality in sepsis. Among complications, SAE and SIC had the strongest evidence and best performance, whereas SAD showed limited utility. Evidence for long-term mortality was extremely scarce. These findings support the potential role of machine learning in early prognostic risk stratification, yet they also highlight the urgent need for rigorous external validation and higher-quality studies, particularly for under-investigated complications.

## 1. Introduction

As revised by the Sepsis-3.0 consensus, sepsis is defined as life-threatening organ dysfunction caused by a dysregulated host response to infection [1]. To date, it remains a leading cause of death among patients in intensive care units (ICUs). Recent global estimates indicate that there were approximately 21.4 million sepsis-related deaths worldwide in 2021, accounting for nearly one-third of all global deaths [2]. The pathophysiology of sepsis involves a complex interplay among invading pathogens, host immune responses, inflammatory cascades, coagulation disorders, microcirculatory dysfunction, metabolic reprogramming, and multi-organ injury [3,4], which further accelerates progression to septic shock, progressive organ failure, and death [5]. It is well documented that early screening, diagnosis, and intervention in septic patients can significantly reduce mortality and optimize patient outcomes [6–8]. Blood cultures are an important tool for diagnosis, but they have several limitations, including relatively low sensitivity and long turnaround times [9]. Studies have shown that in adult patients with septic shock, administering effective antibiotics within one hour of hypotension detection improves in-hospital survival [10]. Therefore, there is an urgent need for accurate and timely prognostic tools to identify high-risk patients and guide individualized treatment strategies.

In recent years, machine learning has emerged as a prominent research direction, leveraging big data analytics to integrate clinical data, biomarker profiles, and imaging features from patients with sepsis and its associated complications, thereby constructing early prediction models that assist clinicians in achieving accurate and rapid diagnosis [11]. Compared with traditional scoring systems such as the Simplified Acute Physiology Score II (SAPS II), the Acute Physiology and Chronic Health Evaluation II scoring system (APACHE II), the Sequential Organ Failure Assessment (SOFA), and the quick Sequential Organ Failure Assessment (qSOFA), machine learning is better equipped to handle dynamically changing patient data [12–15]. By identifying key variables that significantly influence mortality at an early stage, machine learning enhances the efficiency and effectiveness of clinical decision-making.

Zhang et al. conducted a systematic review and meta-analysis of 50 studies to evaluate the performance of machine learning in predicting sepsis-related mortality [16]. Machine learning demonstrated superior predictive performance compared with traditional clinical scoring systems, with random forest (RF) and extreme gradient boosting (XGBoost) identified as the preferred models. However, the study restricted its endpoint to mortality in patients with sepsis and did not further investigate the predictive performance of machine learning for mortality in patients with sepsis-related complications within the evidence synthesis. Moreover, the lack of standardized sepsis definitions and unified study designs precluded effective mitigation of substantial inter-study heterogeneity. Based on this background, this study aims to comprehensively evaluate the performance of machine learning models in predicting mortality in patients with sepsis and its associated complications through a systematic review and meta-analysis. To control heterogeneity, we will restrict the study population, study design, data sources, and mortality observation periods, and conduct subgroup analyses by machine learning algorithm type to identify optimal modeling strategies. The evidence generated from this study is expected to provide a reference for the development and clinical application of machine learning-based prognostic models in patients with sepsis and its associated complications.

## 2. Methods

The protocol for this study was conducted in accordance with the Preferred Reporting Items for Systematic Reviews and Meta-Analyses (PRISMA) guidelines (Supplementary Table S1 in S1 File) [17]. This systematic review and meta-analysis has been registered and approved with the International Prospective Register of Systematic Reviews (PROSPERO) under registration number CRD420261380358.

### 2.1 Search strategy

The search spanned from database inception to April 8, 2026, and was conducted across PubMed, Embase, Cochrane Library, and Web of Science. The search strategy combined Medical Subject Headings (MeSH) and free-text terms, with no geographical restrictions and a language limitation to English. Screening was performed independently by two reviewers, and any disagreements were resolved by a third reviewer. The primary search terms included “machine learning,” “sepsis,” “septic shock,” “sepsis associated encephalopathy,” “sepsis associated acute kidney injury,” “sepsis induced coagulopathy,” “sepsis associated delirium,” “sepsis induced cardiorenal syndrome,” “sepsis induced cardiomyopathy,” “sepsis induced myocardial injury,” and “mortality.” Additionally, a manual “snowballing” search was conducted to identify relevant studies from the reference lists of retrieved articles (Supplementary S2 Table in S1 File).

### 2.2 Inclusion criteria

Studies meeting the following criteria were included: (1) participants aged ≥18 years; (2) studies involving patients diagnosed with sepsis according to the Sepsis-3.0 criteria [1]; (3) sepsis-associated complications, including septic shock, sepsis-associated encephalopathy (SAE), sepsis-associated acute kidney injury (SAKI), sepsis-induced coagulopathy (SIC), sepsis-associated delirium (SAD), sepsis-induced cardiorenal syndrome (SCRS), sepsis-induced cardiomyopathy (SICM), and sepsis-induced myocardial injury (SIMI); (4) study design of cohort studies, including both prospective and retrospective cohorts; (5) studies that developed machine learning prediction models, including both conventional machine learning algorithms and deep learning algorithms, and reported model performance metrics (e.g., sensitivity, specificity, area under the curve [AUC]) or provided sufficient data to derive a confusion matrix; (6) the predicted outcome was mortality, with short-term mortality including in-hospital mortality, ICU mortality, 28-day mortality, 30-day mortality, and 90-day mortality, and long-term mortality including 1-year mortality.

### 2.3 Exclusion criteria

We excluded studies that met the following criteria: (1) studies with insufficient data or those that did not report model performance; (2) studies with cross-sectional, case-control, randomized controlled trial, meta-analysis, review, or guideline study designs; (3) studies with a sample size of fewer than 50 participants; (4) studies that only explored risk factors without establishing a complete prediction model; and (5) studies that focused solely on validated scoring systems.

### 2.4 Study selection and data extraction

Records retrieved from the databases were imported into EndNote X21, and duplicates were removed. Titles and abstracts were screened to exclude ineligible studies. Subsequently, full texts were downloaded and reviewed to determine eligibility. If the two reviewers failed to reach consensus on the final inclusion of a study, a decision was made after consultation with a third reviewer.

Subsequently, we developed a standardized data extraction form based on the following characteristics: first author, year of publication, country, study design, study population, data source, sample size, risk factors, study outcomes, age, imputation method, predictor selection, model type, and calibration indicators. The datasets were defined as follows: the training set was the dataset used to develop or train the machine learning model and estimate its parameters; the internal validation set was a subset of the same source cohort that was not used for training; and the external validation set was an independent dataset from a different population, institution, or geographic region. In the meta-analysis, the reported performance metrics were categorized into two groups: “training set” and “validation set.” Because both internal and external validation sets, although derived from different populations, represent model performance on data not used for training, they were classified under the “validation set” category. If the two reviewers failed to reach consensus on data extraction, a decision was made after consultation with a third reviewer.

### 2.5 Quality assessment

We assessed the methodological quality of the included studies using the Prediction model Risk of Bias Assessment Tool (PROBAST) [18]. Risk of bias was evaluated across four domains—participants, predictors, outcome analysis, and statistical analysis—while applicability was assessed across three domains: participants, predictors, and outcomes. If the two reviewers failed to reach consensus, a decision was made after consulting a third reviewer.

### 2.6 Statistical analysis

All meta-analyses were conducted using STATA 15.0. The primary outcome measures were the AUC, sensitivity, and specificity of the prognostic models. For studies that did not report the AUC with corresponding 95% confidence intervals (CIs) or standard errors (SEs), these values were derived using the formula established by Hanley and McNeil, based on the reported sample size and event rate [19]. To ensure normality and stabilize variance, reported AUC values (expressed as percentages) were converted to proportions and then logit-transformed. Heterogeneity was assessed using Cochran’s Q test and quantified by the I^2^ statistic. A random-effects model was applied when I^2^ > 50%; otherwise, a fixed-effects model was used. An AUC between 0.7 and 0.9 indicated moderate predictive accuracy, while an AUC greater than 0.9 indicated high predictive accuracy. A p-value < 0.05 was considered statistically significant. In addition, a bivariate mixed-effects model was used to conduct meta-analyses of sensitivity and specificity [20]. To further reduce heterogeneity, pre-specified subgroup analyses were performed for each outcome according to study population, validation type, and prognostic outcome. To assess the robustness of the pooled estimates, a “leave-one-out” sensitivity analysis was conducted by sequentially excluding each study and recalculating the pooled effect sizes and I^2^ values, thereby evaluating the influence of individual studies on the overall estimate. For outcomes with 10 or more included studies, publication bias was assessed using funnel plots and Egger’s test [21].

## 3. Results

### 3.1 Study selection

A total of 9,550 records were identified in the initial search. After screening titles and abstracts, 1,050 records were selected for further assessment, of which 909 were excluded. The full texts of the remaining 141 records were sought, and 6 were excluded because the full text could not be obtained. The remaining 135 full-text articles were assessed for eligibility, and 72 studies were ultimately included in the final analysis [22–93]. The detailed screening process is presented in Fig 1.

**Fig 1:**
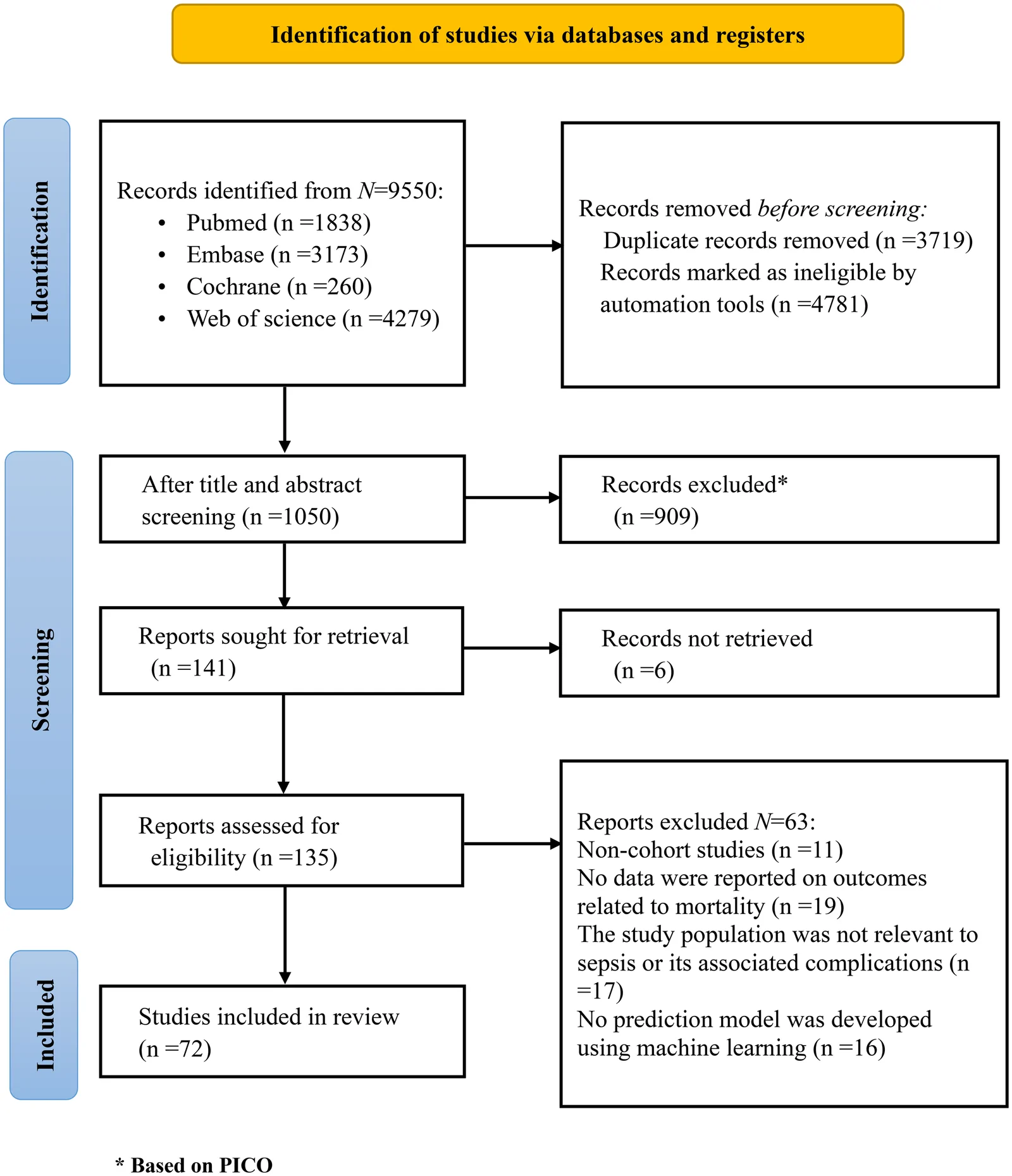
Literature screening process.

### 3.2 Study characteristics

A total of 72 studies were included, comprising 484826 patients, all diagnosed with sepsis and its related complications according to Sepsis-3.0 criteria. Of these, 54 focused on sepsis, 5 on SAKI, 1 on SCRS, 1 on SICM, 1 on SIMI, 1 on septic shock, 1 on SAD, 4 on SAE, and 4 on SIC. All included studies were cohort studies published between 2020 and 2026. Most studies were conducted in China (n = 67), with 1 study each in Italy, Poland, South Korea, and Turkey. The most frequently reported predictors were age (n = 45), lactate (n = 35), heart rate (n = 23), SOFA score (n = 22), respiratory rate (n = 21), blood urea nitrogen (n = 20), white blood cell count (n = 19), platelet count (n = 15), systolic blood pressure (n = 13), and body temperature (n = 13). The majority of studies reported short-term mortality (n = 71), while only 1 study reported long-term mortality. Detailed baseline characteristics of the included studies are presented in Table 1.

**Table 1:** Basic characteristics of the included studies.

First author, year	Country	Research subject	Dataset Area	Total number of samples	Number of samples in the training cohort	Generation of the validation cohort	Number of samples in the validation cohort	Final predictors included (n)	Study outcome
AnShuoyan2025	China	sepsis	eICU, MIMIC-IV	4686	3280	Random sampling	3718	20 (min arterial oxygen saturation [SpO_2_], mini systolic blood pressure [SBP], max lactate, mean lactate, length of hospital stay, min white blood cell [WBC], min heart rate [HR], mean WBC, mean SBP, min mean arterial pressure [MBP], min lactate, min phosphate, length of ICU stay, min platelet, max platelet, maxi sodium, mean MBP, mean phosphate, mean sodium, max HR)	In-hospital mortality
ChenDaonan2024	China	sepsis	MIMIC-IV vasopressor use, mechanical ventil	17329	12130	Random sampling	5199	6 (age, gender, Charlson Comorbidity Index, SOFA score, and APSII score, with the addition of ALC on day 7)	90-day mortality
ChengChiyung2022	China	sepsis	Single-center	193646	116188	Not reported	77458	7 (age, sex, SBP, diastolic blood pressure [DBP], HR, respiratory rate [RR], body temperature [BT])	In-hospital mortality
ChenLinmu2026	China	sepsis	Single-center	1605	1124	Random sampling	481	7 (Glasgow Coma Scale [GCS] score; min pH; max blood urea nitrogen [BUN]; minimum WBC; min platelet; max lactate level; presence of pneumonia)	In-hospital mortality
ChenXianyuan2026	China	sepsis	Single-center	324	Not reported	Not reported	Not reported	6 (number of organ dysfunctions, ICU length of stay, SOFA score, APACHE II score, NPS, SII)	28-day mortality
DrăgoescuAlicenicoleta 2026	Romania	sepsis	Single-center	114	Not reported	Not reported	Not reported	4 (CPR, PLR, PWR, Platelet distribution width to count ratio [PCR])	In-hospital mortality
GanZiyan2026	China	sepsis	Single-center	2152	1722	Random sampling	430	10 (troponin T, platelet distribution width [PDW], neutrophil count [NEUT], red cell distribution width [RDW], fibrinogen [FIB], prothrombin time activity [PTA%], aspartate aminotransferase [AST], urea, low-density lipoprotein cholesterol [LDL-C], high-sensitivity C-reactive protein [hs-CRP])	In-hospital mortality
GaoQiang2025	China	sepsis	Single-center	203	Not reported	Not reported	Not reported	4 (acute respiratory failure, septic shock, chronic liver disease, lactate)	In-hospital mortality
GuoQirui2024	China	sepsis	Single-center	645	387	Random sampling	258	In-hospital mortality: 5 (age, peripheral perfusion index [PI], MAP, SCr, SOFA score) 28-day mortality:5 (peripheral perfusion index [PI], MAP, WBC, APACHE II score, serum urea)	In-hospital mortality, 28-day mortality
HanKun2025	China	sepsis	MIMIC-IV	2032	Not reported	Not reported	Not reported	5 (age, NLR, lactate, mechanical ventilation, qSOFA score)	28-day mortality
HoangQuangvan2025	China	sepsis	Single-center	170	Not reported	Bootstrap resampling	Not reported	4 (LAR, age, respiratory infection, SOFA score)	28-day mortality
HongChengying2025	China	sepsis	Single-center	986	739	Random sampling	247	4 (mechanical ventilation, drug-resistant bacterial infection, hemofiltration, RDW-CV)	In-hospital mortality
HuangYuwei2025	China	sepsis	MIMIC-IV	1965	1376	Random sampling	589	6 (platelet slope, CCI Score, age, ALC mean (7d), ALC recovery ratio, additional clinical features)	90-day mortality
HuChang2022	China	sepsis	MIMIC-IV	8817	7054	Random sampling	1763	6 (GCS score, BUN, RR, urine output, age, anion gap [AG])	In-hospital mortality
HuXuemei2025	China	SAE	eICU, MIMIC-IV	8345	5242	Not reported	3103	8 (BUN, activated partial thromboplastin time [APTT], SpO_2_, HR, Charlson Comorbidity Index [CCI], lactate, vasopressor use, mechanical ventilation)	ICU mortality
JinGuangyong2024	China	SAE	MIMIC-IV	3882	2751	Random sampling	1131	16 (malignant cancer, CCI score, weight, max HR, mean RR, mean temperature, first day urine output, maximum hemoglobin, mean AG, max PTT, min PT, max lactate, min lactate, min PaCO_2_, min GCS score, supplemental oxygen therapy)	1-year mortality
JinJun2026	China	SAKI	eICU, MIMIC-IV	3662	Not reported	Not reported	Not reported	7 (urinary tract infection, catheter-related infection, lactate, dopamine use, norepinephrine use, ICU length of stay [LOS], BUN)	In-hospital mortality
LiCaifeng2024	China	SAKI	MIMIC-IV	1663	1164	Random sampling	499	8 (age, MAP, RR, Lactate, Cr, Prothrombin time-international normalized ratio [PT-INR], Total bilirubin [TBIL], Central venous pressure [CVP])	In-hospital mortality
LiHaitao2023	China	sepsis	Single-center	199	99	Random sampling	100	3 (SOFA score, age, PT)	28-day mortality
LiQiujing2023	China	sepsis	Single-center	464	Not reported	Not reported	Not reported	6 (skeletal muscle index [SMI], cognitive impairment, frailty, HR, RDW, BUN)	In-hospital mortality
LiuTing2026	China	SIMI	MIMIC-IV	7309	5847	Random sampling	1462	15 (vasopressor use, lactate, continuous renal replacement therapy [CRRT], diabetes, age, RR, SpO_2_, acut_2_e kidney injury [AKI], septic shock, coronary artery disease [CAD], AG, WBC, creatinine, troponin T, hypertension)	ICU mortality
LiuXuhui2024	China	sepsis	MIMIC-IV	9943	4585	Random sampling	1966	15 (GCS score, age, SpO_2_, BUN, international normalized ratio [INR], chloride, temperature, RR, partial thromboplastin time [PTT], AG, WBC, hypertension, diabetes, septic shock, antibiotic treatment)	In-hospital mortality
LiuYiguo2022	China	SCRS	Single-center	340	238	External validation	103	5 (age, SOFA score, serum myoglobin [MYO] on day 3, vasopressor use, mechanical ventilation)	1-year mortality
LiXiang2026	China	SICM	MIMIC-IV, Single-center	1068	748	Random sampling	320	7 (Acute Physiology Score III [APS III], age, Cerebrovascular Disease-based on CCI definition, CCI, Alkaline Phosphatase [ALP], lactate, Creatine Kinase-MB[CK-MB])	28-day mortality
LiYongkai2023	China	sepsis	Single-center	382	274	Random sampling	108	5 (shock, age, lactate, lactate-to-albumin ratio, Interleukin-6 [IL-6])	28-day mortality
LiYujie2026	China	sepsis	MIMIC-IV	470	330	Random sampling	140	3 (age, SOFA score, urine output)	28-day mortality
LuoXiang2025	China	sepsis	MIMIC-IV	3364	2355	Random sampling	1009	11 (APSIII, SOFA score, HR, RR, Weight, SpO_2_, ALP, SBP, cerebrovascular diseases, acquired immunodeficiency syndrome [AIDS], chronic obstructive pulmonary disease [COPD])	ICU mortality
LuoXiaoqin2022	China	SAKI	eICU, MIMIC-IV	15603	6066	Random sampling	Internal validation: 3639External validation: 3471	29 (hours from ICU admission, SBP, DBP, HR, RR, BT, SpO_2_, GCS score, urine output, hemoglobin, WBC, platelets, total bilirubin, albumin, SCr, BUN, arterial pH, PaO_2_, PaCO_2_, serum sodium, potassium, chloride, bicarbonate, lactate, INR, PTT, mechanical ventilation, vasopressors, RRT, loop diuretics)	In-hospital mortality
LuoYali2025	China	sepsis	Single-center	31	Not reported	Random sampling	Not reported	3 (bacteroides salyersiae, NK cells in CD45 ⁺ leukocytes, CRP)	28-day mortality
MaChengyong2023	China	sepsis	MIMIC-IV	1584	1108	Random sampling	476	9 (admission type, CCI, APS III, GCS, Serum chloride, RR, SpO_2_, metastatic cancer, moderate-to-severe AKI)	In-hospital mortality
MaXia2025	China	SAKI	MIMIC-IV	12268	8588	Random sampling	3680	45 (the minimum and maximum values of SBP, DBP, HR, temperature, PT, PTT, serum potassium, serum calcium, serum sodium, pH, lactate, WBC, Hb, Platelet count [PLT], and BUN, as well as baseline SCr, tumor, age, AKI stage, glucose fluctuation range, CV, diabetes mellitus, CKD, CHF, moderate/severe liver disease, and cerebrovascular disease, CRRT, mechanical ventilation, statin use, and aspirin administration)	28-day mortality; 90-day mortality
OkoyeChukwuma2025	Italy	sepsis	Single-center	93	Not reported	Not reported	Not reported	9 (age, sex, WBC, platelet count, SCr, hs-CRP, lactate, 50-item Frailty Index [FI], multidrug-resistant [MDR] bacteria status)	In-hospital mortality
PengLiwei2022	China	SAE	MIMIC-IV	6994	4986	bootstrap resampling	2098	15 (APSIII, GCS, SOFA, CCI, RDW, BUN, age, RR, PaO_2_, Temperature, LactateCreatinine, Malignant cancer, Metastatic solid tumor, PLT)	30-day mortality
PlutaMichałP2024	Poland	septic shock	Single-center	55	Not reported	Not reported	Not reported	3 (APACHE II score, CRP concentration, S100B protein concentration)	ICU mortality
QinHongmei2025	China	sepsis	Single-center	338	229	temporal split	109	4 (SOFA score, DIC score, WBC, IL-6)	28-day mortality
ShenLi2025	China	sepsis	eICU	4564	3194	Random sampling	1370	17 (age, admission weight, RR, GCS score, SOFA score, APACHE score, Congestive heart failure [CHF], BUN, ALP, Glucose, AST, total bilirubin, total protein, albumin, lactate, RDW, WBC)	28-day mortality
SheXiao2025	China	sepsis	eICU	13717	6397	temporal split	7320	11(RDW, SOFA score, lactate, pH, 24-hour urine output, PLT, total protein, temperature, HR, GCS score, WBC)	28-day mortality
SongJuhyun2020	South Korea	sepsis	Single-center	160	Not reported	Not reported	Not reported	4 (Pentraxin 3 [PTX3], IL-6, Procalcitonin [PCT], Lactate)	28-day mortality
SuLongxiang2021	China	sepsis	Single-center	2224	1557	Random sampling	667	25 (age, perfusion index, P(v-a)CO_2_/C(a-v)O_2_, pCO_2_, noradrenaline dosage, adrenaline dosage, invasive blood pressure, central venous pressure, P(v-a)CO_2_, sO_2_, lactic acid, invasive SBP, invasive DBP, oxygenation index, white blood cell count, platelet count, total bilirubin, GCS score, creatinine, oxygen concentration, SpO_2_, pO_2_, HR, body temperature, RR)	In-hospital mortality
SunYi2025	China	sepsis	Single-center	613	429	Random sampling	184	6 (PCR, mean platelet volume [MPV], N-terminal proB-type natriuretic peptide [NT-proBNP], lactate, respiratory tract infection, diabetes)	28-day mortality
TanRuimin2025	China	SIC	Single-center	96	Not reported	Not reported	Not reported	3 (PCT, Lactate, SOFA score)	28-day mortality
UnalCetinEce2025	Turkey	sepsis	Single-center	130	Not reported	Not reported	Not reported	3 (Lactate-to-Albumin ratio [L/A ratio], Modified Early Warning Score [MEWS], qSOFA)	30-day mortality
WangQinxin2025	China	sepsis	Single-center	141	Not reported	bootstrap resampling	Not reported	6 (age ≥ 67 years, SOFA score ≥ 7.5, PCT ≥ 5.7 ng/mL, Lactate ≥ 3.5 mmol/L, right ventricular free wall strain [RV-FWS] ≥ –19.4%, RV-GS/PASP ratio ≥ –0.55)	28-day mortality
WangXiaoqian2024	China	sepsis	Multicenter	451	331	Not reported	120	4 (mechanical ventilation, oxygenation index [PaO_2_/FiO_2_ ratio], lactate, BUN])	28-day mortality
WangYiping2025	China	sepsis	MIMIC-IV	27624	18994	Random sampling	Internal validation: 8140External validation: 487	20 (age, BUN, AG, lactate dehydrogenase [LDH], RR, Prothrombin time [PT], Alkaline phosphatase [ALP],24 h ventilation, HR, average increase in Creatinine, glucose, platelets, WBC, temperature, International normalized ratio [INR], SAPS II, PaO_2_/FiO_2_ ratio [P/F ratio], bicarbonate, albumin, CRP)	In-hospital mortality
WeiJian2024	China	sepsis	MIMIC-IV	1251	875	Random sampling	376	4 (GCS score, WBC, RDW, invasive ventilation)	In-hospital mortality
WeiJuan2025	China	sepsis	MIMIC-IV	7532	6025	Random sampling	754	53 (age, sex, height, weight, body Mass Index [BMI], race, myocardial infarction, congestive heart failure, peripheral vascular disease, cerebrovascular disease, dementia, chronic pulmonary disease [COPD], rheumatic disease, peptic ulcer disease, liver disease, diabetes, hemiplegia or paraplegia, renal disease, malignancy, metastatic solid tumor, HR, SBP, DBP, MBP, RR, temperature, SpO_2_, hematocrit, hemoglobin, platelet count, WBC, albumin, AG, bicarbonate, calcium, chloride, BUN, bilirubin, creatinine, glucose, sodium, potassium, INR, PT, PTT, lactate,urine output, GCS, Systemic Inflammatory Response Syndrome [SIRS], APS III, Logistic Organ Dysfunction Score [LODS], Oxford Acute Severity of Illness Score [OASIS], Simplified Acute Physiology Score II [SAPS II], total SOFA score)	28-day mortality; 90-day mortality;1 year mortality
WengLulu2026	China	sepsis	Single-center	407	284	Random sampling	123	7 (age, abdominal infection, vasopressor requirement, WBC, B-type natriuretic peptide [BNP], APACHE II score, mechanical ventilation)	In-hospital mortality.
WuHongsheng2024	China	sepsis	Single-center	582	408	Random sampling	174	11 (Coronary heart disease [CHD], Lymphocyte count [LYMP], Neutrophil-lymphocyte ratio [NLR], RDW, Lactic acid, PT, CRP, PCT, Total bilirubin [Tbil], ALT, IL-6)	In-hospital mortality
WuJinmei2026	China	SIC	MIMIC-IV, Single-center	4505	2797	Random sampling	Internal validation: 700External validation: 133	17 (age, SOFA score, vasopressor use, cardiotonic use, ICU stay time, Creatinine, PT, Total carbon dioxide [TCO_2_], Lactate, AG, Sodium, RDW, platelet count, SBP, Type 2 diabetes, heart failure, acute renal failure)	28-day mortality
XieYinjing2023	China	sepsis	Single-center	367	257	Random sampling	110	5 (IL-6D1 [IL-6 level within 24 hours of admission], IL-6D3 [IL-6 level at 48–72 hours], PCTD1[PCT level within 24 hours], PCTD3 [PCT level at 48–72 hours], and LACcD3)	28-day mortality
XinQi026	China	sepsis	Single-center	635	477	Random sampling	158	7 (albumin-alkaline phosphatase ratio [AAPR], albumin-bilirubin grade [ALBI], neutrophil-lymphocyte ratio [NLR], platelet-to-lymphocyte ratio [PLR], prognostic nutritional index [PNI], systemic immune-inflammation index [SII], and lactate-albumin ratio (LAR))	28-day mortality
XinQi2026a	China	sepsis	Single-center	246	185	Random sampling	61	6 (hepatic encephalopathy [HE], D-dimer [D-D], platelet-to-lymphocyte ratio [PLR], lymphocyte-to-monocyte ratio [LMR], ALBI grade (albumin-bilirubin grade), systemic inflammation response index [SIRI])	28-day mortality
XuXin2026	China	sepsis	eICU, MIMIC-IV	1615	1130	Random sampling	Internal validation: 485External validation: 325	6 (age, WBC, total bilirubin, glucose, INR, lactate)	28-day mortality
XuYaya2022	China	SAKI	eICU, MIMIC-IV	14783	9170	Not reported	5613	17 (age, sex, SOFA score, APS-III score, Charlson comorbidity index, use of mechanical ventilation, use of RRT, use of vasopressors, MAP, SV, Lac, BUN, SCr, NLR, mean perfusion pressure [MPP], initial CVP level, and change in CVP within 48 hours [ΔCVP])	28-day mortality
XuYifei2026	China	sepsis	Single-center	364	Not reported	Not reported	Not reported	12 (SII, APACHE II score, Pneumonia Severity Index [PSI], CURB-65, SOFA score, ALB, PNI, age, PCT, CAR, Scr, and gender)	28-day mortality
XuZhikun2026	China	sepsis	eICU, MIMIC-IV, Single-center	2052	1328	Not reported	724	13 (age, RR, BT, SpO_2_, HR_2_, total bilirubin, lactate, creatinine, WBC, INR, vasopressor use, urine output (< 400 ml), GCS score)	In-hospital mortality
YangHao2025	China	sepsis	MIMIC-IV, Multicenter	3461	2758	Random sampling	Internal validation: 703External validation: 2173	42 (age, gender, BMI, cardiovascular disease, hypertension, chronic kidney disease, chronic respiratory disease, autoimmune disease, PCT, CRP, eosinophil, neutrophil count, globulins, autoimmune disease, anion gap, troponin-T, myoglobin, bilirubin, sodium, calcium, lactic acid, HBDH, glucose, triglycerides, INR, impedance platelet, RDW, temperature, HR, blood pressure, SpO_2_, PaCO_2_, and combined markers (age+autoimmune disease, age + PCT, age + PaCO_2_)	In-hospital mortality
YangJianyue2025	China	SIC	MIMIC-IV, Single-center	4554	Not reported	Random sampling	Internal validation: 911External validation: 367	8 (age, RDW, mean corpuscular volume [MCV], lactate, APSIII, log_2_-transformed pan-immune-inflammation value [PIV], continuous renal replacement therapy [CRRT], AKI)	In-hospital mortality
YangMeicheng2024	China	sepsis	eICU, MIMIC-IV	20933	16746	Random sampling	4187	15 (APS III score, age, urine output, vasopressors, pulmonary infection, lactate (min), BMI, WBC, SpO_2_, max platelet count, mean RR, min AG, min BUN, max aPTT, and max serum sodium).	In-hospital mortality
YangYang2022	China	SAE	MIMIC-III	2474	1732	Random sampling	742	7 (age, lactate, bilirubin, RDW, mean RR (min ^−1^), mean temperature (°C), use of vasopressor)	30-day mortality
YangYanjie2025	China	sepsis	Multicenter	822	580	Not reported	242	5 (age, SOFA score, CRP, mechanical ventilation, vasoactive drugs)	28-day mortality
YangYong2022	China	sepsis	Single-center	317	229	Random sampling	88	4 (NLR d3, BNP d3, fluid accumulation at 72 hours, and SOFA score)	28-day mortality
YinJingjing2026	China	SAD	MIMIC-IV	5390	Not reported	Random sampling	1617	15 (BUN, age, PT, weight, temperature, PTT, platelets, stroke, hemoglobin, RR, chloride, SpO_2_, HR, glucose, SBP)	30-day mortality
YuXiaoyuan2025	China	sepsis	MIMIC-IV	501	376	Random sampling	125	8 (age, HR, RR, BUN, creatinine [Cr], lactate, pH, and urine output)	28-day mortality
Zeng Zhixuan 2021	China	sepsis	MIMIC-III, eICU-CRD	24653	12558	External validation	12095	34 (age, BMI, admission type, AIDS, hematological tumor, metastatic cancer, HR, RR, temperature, SBP, MAP, SpO_2_, PaO_2_, PaCO_2_, PaO_2_FiO_2_, WBC, platelets, hemoglobin, bilirubin, albumin, creatinine, BUN, sodium, potassium, lactate, PT, GCS, mechanical ventilation, RRT, and 24h urine output)	In-hospital mortality
ZengQingbo2021	China	sepsis	Single-center	231	161	Random sampling	70	4 (age [≥57 years vs. < 57 years], international normalized ratio [INR], lactate, and thromboembolism [TM] level)	90-day mortality
ZhangDalong2025	China	sepsis	Single-center	81	Not reported	bootstrap resampling	Not reported	6 (PCT, CRP, lactate, LCR, TLR4 rs4986790 [AG genotype], PPARγ rs1801282 [CG genotype])	28-day mortality
ZhangGuyu2024	China	sepsis	eICU, MIMIC-IV, AmsterdamUMCdb	3535	1696	Random sampling	1239	12(age, HR, AST, invasive ventilation treatment, renal replacement treatment, albumin, cerebrovascular disease, monocyte-to-HDL ratio [MHR], NLR, NHR, BUN, Potassium)	In-hospital mortality
ZhangJing2026	China	sepsis	Single-center	578	404	Random sampling	174	7 (age, respiratory failure within 48 hours, multiple organ dysfunction syndrome [MODS] within 48 hours, INR, lactate, prognostic nutrition index [PNI], and triglyceride-glucose [TyG] index)	28-day mortality
ZhouShu2024	China	SIC	MIMIC-III, MIMIC-IV, eICU	3280	2296	Random sampling	Internal validation: 984External validation: 4466	17 (age, gender, SOFA score, mean HR, MAP, mean RR, temperature, max lactate, min platelet count, max PTT, max PT, max BUN, max WBC, min PO_2_, min MCHC, max RDW, min MCV)	28-day mortality
ZhouXuan2025	China	sepsis	MIMIC-IV	17226	12058	Random sampling	5168	6 (CCI, APS III score, LODS score, mean RR, mean BT, race)	In-hospital mortality

*Note: SAD: sepsis-associated delirium; SAE: sepsis-associated encephalopathy; SAKI: sepsis-associated acute kidney injury; SCRS: sepsis-induced cardiorenal syndrome; SIC: sepsis-induced coagulopathy; SICM: sepsis-induced cardiomyopathy; SIMI: sepsis-induced myocardial injury*

Details on the predictive models used in the included studies are presented in Table 2. For missing data, multiple imputation was the most commonly used strategy (n = 29). For variable selection, 31 studies reported using LASSO regression, and 29 employed univariate analysis combined with multivariate logistic regression. A total of 28 machine learning models were included, comprising 22 traditional machine learning algorithms (Adaptive Boosting [AdaBoost], Classification and Regression Tree [CART], Categorical Boosting [CatBoost], Complement Naive Bayes [CNB], Gradient Boosting Machine + Conformal Prediction [CPMORS], Decision Tree [DT], Explainable Boosting Machine [EBM], Elastic Net [EN], Gradient Boosting Tree [GBT], GBM [Gradient Boosting Machine], K-Nearest Neighbors [KNN], Least Absolute Shrinkage and Selection Operator [LASSO], Light Gradient Boosting Machine [LightGBM], Linear Discriminant Analysis [LDA], Logistic Regression [LR], Naive Bayes [NB], Neural Decision Forest [NDF], Random Forest [RF], Random Survival Forests [RSF], Stochastic Gradient Boosting [SGBT], Support Vector Machine [SVM], and eXtreme Gradient Boosting [XGBoost]) and 6 deep learning algorithms (Convolutional Neural Networks [CNN], Deep Neural Networks [DNN], Long Short-Term Memory [LSTM], Multilayer Perceptron [MLP], Neural Network [NNET], and Deep Learning based Combined Model [DL-CMT]). The most frequently used machine learning model was LR (n = 63), followed by RF (n = 22) and SVM (n = 19).

**Table 2:** Functions of mortality prediction models for sepsis and its associated complications.

First author, year	Imputation method	Predictor selection	Model type	Calibration indicators
AnShuoyan2025	Multiple Imputation	Boruta algorithm	LR, DT, KNN, RF, SVM, NNET, GBT, XGBoost	Calibration curves, DCA
ChenDaonan2024	Forward fill method	Not reported	DT, GBT, LR, SVM, RF, XGBoost	Not reported
ChengChiyung2022	Forward fill method	Not reported	CNN, LSTM, RF	Not reported
ChenLinmu2026	Multiple Imputation	Univariate and multivariate logistic regression; Boruta algorithm; LASSO regression	LR, SVM, RF, XGBoost, KNN, MLP	Hosmer-Lemeshow test, Calibration plot, Brier score
ChenXianyuan2026	Not reported	Univariate and multivariate logistic regression	LR	Calibration curves, DCA
DrăgoescuAlicenicoleta 2026	Not reported	Univariate and multivariate logistic regression	LR	Not reported
GanZiyan2026	Multiple Imputation	Recursive Feature Elimination	LR, DT, RF, KNN; SVM, NB, SGBT, NNET	Calibration plot
GaoQiang2025	Mean imputation	Univariate and multivariate logistic regression	LR	Not reported
GuoQirui2024	Median imputation	Multivariate logistic Regression; LASSO regression	LR	Calibration curves, DCA
HanKun2025	Multiple imputation	Univariate and multivariate logistic regression; LASSO regression	LR	Calibration plot, DCA
HoangQuangvan2025	Not reported	Univariate and multivariate logistic regression; LASSO regression	LR	Calibration plot, brier score, calibration slope
HongChengying2025	Not reported	Multivariate logistic Regression	LR	Calibration plots, DCA
HuChang2022	Multiple imputation	LASSO regression	XGBoost, RF, NB, LR, SVM, KNN, DT	Calibration curve
HuXuemei2025	Multiple imputation	LASSO regression	LR	Calibration curves, Hosmer-Lemeshow goodness-of-fit test, brier score, DCA
HuangYuwei2025	Not reported	SHAP analysis	XGBoost	Calibration curve
JinGuangyong2024	Multiple imputation	Multivariate logistic Regression; LASSO regression	LR	Calibration curves, DCA
JinJun2026	Multiple imputation	Univariate and multivariate logistic regression	LR	Not reported
LiQiujing2023	Multiple Imputation	Multivariate logistic Regression; LASSO regression	LR	Hosmer-Lemeshow goodness-of-fit test; calibration plot
LiHaitao2023	Not reported	Univariate and multivariate Cox regression	LR	Hosmer-Lemeshow test
LiYongkai2023	Not reported	Univariate and multivariate logistic regression	LR	Hosmer-Lemeshow test
LiCaifeng2024	Multiple imputation	LASSO regression; Boruta algorithm	CART, SVM, LR	Calibration curves, Hosmer-Lemeshow test, brier score, DCA
LiXiang2026	Random Forest	Boruta algorithm; LASSO regression; Recursive Feature Elimination with Cross-Validation	XGBoost, RF, AdaBoost, GNB, CNB, SVM, LR, KNN	Calibration curves, brier score, Kolmogorov-Smirnov statistic, DCA
LiYujie2026	Multiple imputation	LASSO regression	LR	Calibration plots, brier scores
LiuYiguo2022	Not reported	Random Forest	SVM, RF, GBT, XGBoost, LR, LightGBM	Calibration curves, brier scores, DCA
LiuXuhui2024	Multiple imputation	LASSO regression	LR, DT, EN, KNN, LightGBM, RF, XGBoost, SVM, MLP	Calibration curves, DCA
LiuTing2026	Multiple imputation	Random Forest; XGBoost; Logistic Regression	LR	Calibration plots, DCA
LuoXiaoqin2022	Not reported	XGBoost	XGBoost	Calibration curves
LuoYali2025	Not reported	LASSO regression	RF	Calibration plots
LuoXiang2025	Multiple imputation	LASSO regression; Recursive Feature Elimination	NB, KNN, DT, RF, XGBoost, SVM.	Brier score, calibration plots
MaChengyong2023	Multiple imputation	Multivariate logistic Regression; LASSO regression	LR	Calibration curves, brier scores, DCA
MaXia2025	Multiple imputation	Boruta algorithm	LASSO, RSF	Calibration curves, DCA, calibration slopes
OkoyeChukwuma2025	Not reported	Univariate and multivariate logistic regression	LR	Calibration plots; Hosmer-Lemeshow goodness-of-fit test; bootstrap resampling
PengLiwei2022	Multiple imputation	Recursive Feature Elimination	NNET, NB, LR, GBM, Ada, RF, BT, XGB, CatBoost.	Hosmer-Lemeshow goodness-of-fit test, Calibration curves
PlutaMichałP2024	Not reported	Univariate and multivariate logistic regression	LR	Hosmer-Lemeshow test
QinHongmei2025	Not reported	Univariate and multivariate logistic regression	LR	Calibration curves, DCA
SheXiao2025	Not reported	Univariate and multivariate logistic regression; Random Forest; LASSO regression	LR	Calibration curves, DCA
ShenLi2025	Not reported	LASSO regression; Boruta algorithm	LR, DT, XGBoost, SVM, LightGBM	Not reported
SongJuhyun2020	Not reported	Not reported	LR	Hosmer-Lemeshow test
SuLongxiang2021	KNN imputation	LASSO regression	LR, RF, XGBoost	Not reported
SunYi2025	Not reported	Univariate and multivariate logistic regression; LASSO regression	LR	Calibration curves, Hosmer-Lemeshow test, DCA
TanRuimin2025	Not reported	Univariate and multivariate logistic regression	LR	Not reported
UnalCetinEce2025	Not reported	Univariate and multivariate logistic regression	LR	Hosmer-Lemeshow test
WangYiping2025	Multiple Imputation	Not reported	LR, SVM, DNN, XGBoost	Not reported
WangXiaoqian2024	Not reported	Univariate and multivariate logistic regression	LR	Calibration curves, DCA
WangQinxin2025	Not reported	Univariate and multivariate logistic regression; LASSO regression	LR	Calibration curves, DCA, Hosmer-Lemeshow test
WeiJian2024	Multiple imputation	LASSO regression	LR	Calibration curves, Hosmer-Lemeshow test
WeiJuan2025	Multiple imputation	Automatic feature extraction	DL-CMT, MLP, RF, XGBoost	Not reported
WengLulu2026	Multiple imputation	Univariate and multivariate logistic regression; LASSO regression	LR	Calibration curves, DCA
WuHongsheng2024	Multiple Imputation	LASSO regression	LR	Hosmer-Lemeshow test, Calibration curves
WuJinmei2026	Multiple imputation	Multivariate logistic Regression; LASSO regression	XGBoost, LR, LightGBM, RF, AdaBoost, MLP, SVM, KNN, CNB	Calibration curves, DCA
XieYinjing2023	Multiple imputation	Univariate linear regression and multivariate stepwise regression	LR	Calibration plots, DCA
XinQi2026a	Not reported	Univariate and multivariate logistic regression; LASSO regression	LR	Calibration curves, DCA
XinQi026	Not reported	Univariate and multivariate logistic regression; LASSO regression	LR	Calibration plots, DCA
XuYaya2022	Not reported	Univariate linear regression and multivariate stepwise regression	LR	Calibration curves
XuXin2026	Multiple imputation	LASSO regression	LR, SVM, RF, GBM, XGBoost, LightGBM, CatBoost, KNN, NB, MLP	Calibration curves, DCA
XuYifei2026	Multiple imputation	Boruta algorithm	GBM, LR, RF, LightGBM, XGBoost	Calibration plots, DCA
XuZhikun2026	Multiple imputation	LASSO regression	LR	Calibration curves, DCA
YangYang2022	Multiple Imputation	Univariate and multivariate logistic regression	LR	Calibration plot, brier score, Hosmer-Lemeshow test
YangYong2022	Not reported	Univariate and multivariate logistic regression	LR	Calibration plots
YangMeicheng2024	Not reported	Not reported	CPMORS [GBM + Conformal Prediction], NDF, RF, LR	Calibration curves
YangYanjie2025	Multiple imputation	Univariate and multivariate logistic regression	LR	Calibration curves, DCA, Hosmer-Lemeshow goodness-of-fit test
YangJianyue2025	Multiple imputation	LASSO regression	DT, RF, NB, SVM, LR, GBM	Calibration curves, DCA
YangHao2025	Forward filling, Median imputation	Not reported	EBM, LSTM	Not reported
YinJingjing2026	Not reported	Boruta algorithm	LR, SVM, DT, RF, XGBoost, KNN, NB	Calibration curves, DCA
YuXiaoyuan2025	Not reported	Univariate and multivariate logistic regression	LR	Calibration curves, DCA
ZengQingbo2021	Not reported	Univariate and multivariate logistic regression	LR	Calibration curves, DCA
ZengZhixuan 2021	KNN imputation	Stepwise logistic regression	LR, LDA, CART, NB, KNN, MLP, SVM, RF, XGBoost	Calibration curves
ZhangGuyu2024	Multiple imputation	LASSO regression	XGBoost, LR, RF, SVM, KNN, NB, DT	Calibration curves, DCA
ZhangDalong2025	Not reported	LASSO regression	LR	Hosmer-Lemeshow test, DCA
ZhangJing2026	Not reported	Univariate and multivariate logistic regression	LR	Calibration curves, DCA
ZhouShu2024	Not reported	Recursive Feature Elimination	LR, SVM, NB, XGBoost	Calibration plots, DCA
ZhouXuan2025	Not reported	Univariate and multivariate logistic regression	LR	Not reported

*Note: AdaBoost: adaptive boosting, CART: classification and regression tree, CatBoost: categorical boosting, CNB: complement naive Bayes, CNN: convolutional neural networks, CPMORS: gradient boosting machine + conformal prediction, DCA: decision curve analysis, DL-CMT: Deep learning-based combined model, DNN: deep neural networks; DT: decision tree, EBM: explainable boosting machine, EN: elastic net, GBT: gradient boosting tree, GBM: gradient boosting machine, GNB: Gaussian naive Bayes, KNN: k-nearest neighbors, LASSO: least absolute shrinkage and selection operator, LightGBM: Light gradient boosting machine, LDA: linear discriminant analysis, LR: logistic regression, LSTM: long short-term memory, MLP: multilayer perceptron, NB: naive Bayes, NDF: neural decision forest, NNET: neural network, RF: random forest, RSF: random survival forests, SGBT: stochastic gradient boosting, SVM: support vector machine, XGBoost: extreme gradient boosting.*

### 3.3 Risk of bias and applicability

The results of the quality assessment using the PROBAST tool showed that among the 72 included studies, 17 were judged to have a high overall risk of bias, while 55 had an unclear overall risk of bias. Domain-specific evaluations indicated that the Participants, Predictors, and Outcome domains were rated as having a low risk of bias, whereas the risk of bias was primarily concentrated in the Analysis domain. Regarding Applicability, 69 studies were rated as having low concern regarding overall applicability, while 3 studies were rated as having high concern, suggesting that the overall applicability of the included studies was satisfactory. Detailed results are presented in Supplementary S3 Table in S1 File.

### 3.4 Meta-analysis

#### 3.4.1 The machine learning model predicted short-term mortality with an AUC value based on the training set.

Among patients with sepsis, 46 studies across 15 model types were included. The overall pooled AUC was 0.82 (95% CI: 0.81–0.83), with substantial heterogeneity (I^2^ = 99.2%). Among the individual models, LR was the most frequently evaluated (n = 27), yielding a pooled AUC of 0.84 (95% CI: 0.80–0.87), although heterogeneity remained significant (I^2^ = 98.6%). RF and XGBoost were each assessed in 3 studies, with pooled AUCs of 0.81 (95% CI: 0.79–0.84, I^2^ = 95.3%) and 0.81 (95% CI: 0.79–0.84, I^2^ = 95.9%), respectively. SVM was evaluated in 2 studies, with a pooled AUC of 0.75 (95% CI: 0.69–0.83, I^2^ = 99.4%). DT, GBT, LightGBM, GBM, EBM, LSTM, MLP, LDA, NB, KNN, and CART were each examined in a single study, with AUCs ranging from 0.73 to 0.92. Among all machine learning models, LightGBM achieved the highest AUC (0.92, 95% CI: 0.88–0.96), albeit based on only one study, whereas CART (AUC = 0.73, 95% CI: 0.73–0.74, I^2^ = 0%) and EBM (AUC = 0.73, 95% CI: 0.70–0.76, I^2^ = 0%) exhibited the lowest AUCs. See Table 3. The pooled sensitivity was 0.79 (95% CI: 0.74–0.83), and the pooled specificity was 0.75 (95% CI: 0.68–0.80). See Supplementary S1 Fig in S1 File.

**Table 3:** Training set–based forest plot of AUC values.

	Short-term mortality				Long-term mortality			
	Model type	Number of studies included	I^2^	AUC (95%CI)	Model type	Number of studies included	I^2^	AUC (95%CI)
**Sepsis**	DT	1	0	0.75 (0.74, 0.76)	–	–	–	–
	GBT	1	0	0.86 (0.85, 0.87)	–	–	–	–
	LR	27	98.6%	0.84 (0.80, 0.87)	–	–	–	–
	SVM	2	99.4%	0.75 (0.69, 0.83)	–	–	–	–
	RF	3	95.3%	0.81 (0.79, 0.84)	–	–	–	–
	XGBoost	3	95.9%	0.81 (0.79, 0.84)	–	–	–	–
	LightGBM	1	0	0.92 (0.88, 0.96)	–	–	–	–
	GBM	1	0	0.91 (0.86, 0.95)	–	–	–	–
	EBM	1	0	0.73 (0.70, 0.76)	–	–	–	–
	LSTM	1	0	0.78 (0.75, 0.81)	–	–	–	–
	MLP	1	0	0.80 (0.79, 0.80)	–	–	–	–
	LDA	1	0	0.79 (0.79, 0.80)	–	–	–	–
	NB	1	0	0.77 (0.77, 0.77)	–	–	–	–
	KNN	1	0	0.76 (0.76, 0.77)	–	–	–	–
	CART	1	0	0.73 (0.73, 0.74)	–	–	–	–
	Overall	46	99.2%	0.82 (0.81, 0.83)	–	–	–	–
**SAE**	LR	2	94.5%	0.79(0.73, 0.87)	LR	1	0	0.88(0.87, 0.90)
	Overall	2	94.5%	0.79(0.73, 0.87)	Overall	1	0	0.88(0.87, 0.90)
**SAKI**	LR	2	88.4%	0.79 (0.67, 0.94)	–	–	–	–
	SVM	1	0	0.79 (0.78, 0.80)	–	–	–	–
	CART	1	0	0.77 (0.74, 0.80)	–	–	–	–
	Overall	2	91.9%	0.78 (0.73, 0.82)	–	–	–	–
**SIC**	RF	1	0	0.84 (0.83, 0.85)	–	–	–	–
	GBM	1	0	0.74 (0.73, 0.75)	–	–	–	–
	SVM	1	0	0.73 (0.72, 0.74)	–	–	–	–
	LR	1	0	0.73 (0.72, 0.74)	–	–	–	–
	NB	1	0	0.69 (0.68, 0.71)	–	–	–	–
	DT	1	0	0.67 (0.66, 0.68)	–	–	–	–
	Overall	1	98.1%	0.73 (0.70, 0.76)	–	–	–	–
**SICM**	LR	1	0	0.82 (0.79, 0.85)	–	–	–	–
	XGBoost	1	0	0.89 (0.86,0.91)	–	–	–	–
	RF	1	0	0.98 (0.97, 0.99)	–	–	–	–
	AdaBoost	1	0	0.85 (0.82, 0.88)	–	–	–	–
	GNB	1	0	0.79 (0.76, 0.83)	–	–	–	–
	CNB	1	0	0.73 (0.68, 0.77)	–	–	–	–
	SVM	1	0	0.79 (0.75, 0.83)	–	–	–	–
	KNN	1	0	0.91 (0.89, 0.93)	–	–	–	–
	Overall	1	97.9%	0.84 (0.79, 0.90)	–	–	–	–

For patients with SAE, a total of 2 studies were included, both using the LR model. The pooled AUC was 0.79 (95% CI: 0.73–0.87), with substantial heterogeneity (I^2^ = 94.5%). See Table 3.

Among patients with SAKI, 2 studies involving 3 model types were included. For LR, evaluated in 2 studies, the pooled AUC was 0.79 (95% CI: 0.67–0.94, I^2^ = 88.4%). SVM was assessed in 1 study, with an AUC of 0.79 (95% CI: 0.78–0.80), and CART was also evaluated in 1 study, with an AUC of 0.77 (95% CI: 0.74–0.80). The overall pooled AUC across all models was 0.78 (95% CI: 0.73–0.82, I^2^ = 91.9%). See Table 3. The pooled sensitivity was 0.78 (95% CI: 0.75–0.80), and the pooled specificity was 0.70 (95% CI: 0.56–0.81). See Supplementary S2 Fig in S1 File.

For patients with SIC, 1 study involving 6 model types was included. RF performed best, with an AUC of 0.84 (95% CI: 0.83–0.85), whereas DT had the lowest AUC of 0.67 (95% CI: 0.66–0.68). The remaining models had AUCs ranging from 0.69 for NB to 0.74 for GBM. The overall pooled AUC across all models was 0.73 (95% CI: 0.70–0.76, I^2^ = 98.1%). See Table 3.

Among patients with SICM, 1 study involving 8 model types was included. RF performed best, with an AUC of 0.98 (95% CI: 0.97–0.99), followed by KNN (AUC 0.91, 95% CI: 0.89–0.93) and XGBoost (AUC 0.89, 95% CI: 0.86–0.91). CNB had the lowest AUC of 0.73 (95% CI: 0.68–0.77). The overall pooled AUC across all models was 0.84 (95% CI: 0.79–0.90, I^²^ = 97.9%). See Table 3. The pooled sensitivity was 0.77 (95% CI: 0.63–0.87), and the pooled specificity was 0.80 (95% CI: 0.68–0.88). See Supplementary S3 Fig in S1 File.

#### 3.4.2 The machine learning model predicted long-term mortality with an AUC value based on the training set.

Long-term mortality was reported only for patients with SAE. A single study used the LR model, yielding an AUC of 0.88 (95% CI: 0.87–0.90). See Table 3.

#### 3.4.3 The machine learning model predicted short-term mortality with an AUC value based on the validation set.

Among patients with sepsis, 50 studies involving 25 machine learning models were included. LR was the most frequently evaluated model (n = 46), with an AUC of 0.81 (95% CI: 0.79–0.83). GBM, EBM, CART, CNN, SGBT, EN, DNN, DL-CMT, CatBoost, CPMORS, and NDF were each assessed in only one study. Across all models, DL-CMT achieved the highest AUC (0.94, 95% CI: 0.91–0.97), whereas KNN yielded the lowest AUC (0.68, 95% CI: 0.62–0.74). The pooled AUC across all models was 0.79 (95% CI: 0.78–0.80). See Table 4. The pooled sensitivity was 0.75 (95% CI: 0.72–0.78), and the pooled specificity was 0.74 (95% CI: 0.71–0.77).

**Table 4:** Validation set set–based forest plot of AUC values.

	Short-term mortality				Long-term mortality			
	Model type	Number of studies included	I^2^	AUC (95%CI)	Model type	Number of studies included	I^2^	AUC (95%CI)
**Sepsis**	DT	8	90.1%	0.69 (0.66, 0.72)	–	–	–	–
	GBT	2	99.9%	0.80 (0.63, 1.01)	–	–	–	–
	LR	46	96.1%	0.81 (0.79, 0.83)	–	–	–	–
	SVM	11	98.8%	0.77 (0.73, 0.81)	–	–	–	–
	RF	15	97.8%	0.81 (0.78, 0.83)	–	–	–	–
	XGBoost	13	99.2%	0.83 (0.79, 0.86)	–	–	–	–
	LightGBM	3	61.1%	0.77 (0.73, 0.82)	–	–	–	–
	GBM	1	0	0.88 (0.81, 0.96)	–	–	–	–
	EBM	1	28.2%	0.69 (0.65, 0.73)	–	–	–	–
	LSTM	2	0.0%	0.74 (0.74, 0.74)	–	–	–	–
	MLP	4	97.6%	0.81 (0.76, 0.86)	–	–	–	–
	LDA	2	92.2%	0.78 (0.76, 0.81)	–	–	–	–
	NB	6	95.1%	0.72 (0.68, 0.77)	–	–	–	–
	KNN	8	98.2%	0.68 (0.62, 0.74)	–	–	–	–
	CART	1	0	0.71 (0.70, 0.72)	–	–	–	–
	NNET	2	0	0.76 (0.74, 0.78)	–	–	–	–
	CNN	1	0	0.83 (0.83, 0.83)	–	–	–	–
	SGBT	1	0	0.83 (0.79, 0.87)	–	–	–	–
	EN	1	0	0.80 (0.77, 0.82)	–	–	–	–
	LGBM	2	62.3%	0.78 (0.72, 0.84)	–	–	–	–
	DNN	1	0	0.84 (0.83, 0.85)	–	–	–	–
	DL-CMT	1	81.7%	0.94 (0.91, 0.97)	–	–	–	–
	CatBoost	1	0	0.74 (0.70, 0.78)	–	–	–	–
	CPMORS	1	97.8%	0.83 (0.77, 0.89)	–	–	–	–
	NDF	1	98.0%	0.82 (0.76, 0.89)	–	–	–	–
	Overall	50	98.6%	0.79 (0.78, 0.80)	–	–	–	–
**SAD**	LR	1	0	0.73 (0.70, 0.77)	–	–	–	–
	SVM	1	0	0.71 (0.67, 0.75)	–	–	–	–
	XGBoost	1	0	0.74 (0.71, 0.78)	–	–	–	–
	RF	1	0	0.53 (0.48, 0.58)	–	–	–	–
	DT	1	0	0.64 (0.59, 0.68)	–	–	–	–
	NB	1	0	0.69 (0.65, 0.73)	–	–	–	–
	KNN	1	0	0.67 (0.62, 0.71)	–	–	–	–
	Overall	1	87.5%	0.67 (0.63, 0.72)	–	–	–	–
**SAE**	LR	3	80.7%	0.81(0.78, 0.85)	LR	1	0	0.86(0.83, 0.89)
	AdaBoost	1	0	0.83(0.82, 0.85)	–	–	–	–
	NNET	1	0	0.83(0.82, 0.85)	–	–	–	–
	LR	1	0	0.83(0.81, 0.85)	–	–	–	–
	XGBoost	1	0	0.83(0.81, 0.85)	–	–	–	–
	CatBoost	1	0	0.83(0.82, 0.85)	–	–	–	–
	RF	1	0	0.83(0.82, 0.85)	–	–	–	–
	GBM	1	0	0.83(0.81, 0.84)	–	–	–	–
	NB	1	0	0.82(0.81, 0.84)	–	–	–	–
	BT	1	0	0.83(0.82, 0.85)	–	–	–	–
	Overall	3	56.7%	0.80(0.79, 0.82)	Overall	1	0	0.86(0.83, 0.89)
**SAKI**	LR	2	26.4%	0.76 (0.74, 0.78)	–	–	–	–
	SVM	1	0	0.71 (0.67, 0.76)	–	–	–	–
	CART	1	0	0.66 (0.62, 0.71)	–	–	–	–
	XGBoost	2	76.8%	0.83 (0.81, 0.86)	–	–	–	–
	RSF	2	0	0.85 (0.83, 0.86)	–	–	–	–
	LASSO	2	0	0.82 (0.81, 0.83)	–	–	–	–
	Overall	5	92.0%	0.79 (0.77, 0.81)	–	–	–	–
**SCRS**	–	–	–	–	RF	1	0	0.83 (0.78, 0.87)
	–	–	–	–	LGBM	1	0	0.80 (0.74, 0.84)
	–	–	–	–	GBT	1	0	0.78 (0.72, 0.82)
	–	–	–	–	XGBoost	1	0	0.71 (0.65, 0.76)
	–	–	–	–	DT	1	0	0.75 (0.69, 0.80)
	–	–	–	–	SVM	1	0	0.68 (0.62, 0.73)
					Overall	1	77.4%	0.76 (0.72, 0.81)
**SIC**	RF	2	38.8%	0.81 (0.79, 0.84)	–	–	–	–
	CNB	1	0	0.64 (0.57, 0.72)	–	–	–	–
	SVM	2	93.1	0.79 (0.75, 0.84)	–	–	–	–
	LR	3	70.0%	0.82 (0.80, 0.84)	–	–	–	–
	NB	1	0	0.80 (0.78, 0.82)	–	–	–	–
	KNN	1	0	0.62 (0.55, 0.69)	–	–	–	–
	XGBoost	2	92.3%	0.88 (0.84, 0.91)	–	–	–	–
	AdaBoost	1	0	0.81 (0.77, 0.85)	–	–	–	–
	MLP	1	0	0.75 (0.70, 0.80)	–	–	–	–
	LGBM	1	0	0.63 (0.57, 0.72)	–	–	–	–
	Overall	4	95.1%	0.80 (0.77, 0.82)	–	–	–	–
**SICM**	LR	1	0	0.82 (0.74, 0.89)	–	–	–	–
	XGBoost	1	0	0.79 (0.71,0.86)	–	–	–	–
	RF	1	0	0.79 (0.72, 0.87)	–	–	–	–
	AdaBoost	1	0	0.78 (0.70, 0.85)	–	–	–	–
	GNB	1	0	0.72 (0.63, 0.81)	–	–	–	–
	CNB	1	0	0.78 (0.69, 0.86)	–	–	–	–
	SVM	1	0	0.78 (0.69, 0.86)	–	–	–	–
	KNN	1	0	0.70 (0.61, 0.78)	–	–	–	–
	Overall	1	0%	0.78 (0.75, 0.81)	–	–	–	–
**SIMI**	LR	1	0	0.77 (0.76, 0.78)	–	–	–	–
	Overall	1	0%	0.77 (0.76, 0.78)	–	–	–	–
**Septic shock**	LR	1	0	0.95 (0.85, 0.99)	–	–	–	–
	Overall	1	0%	0.95 (0.85, 0.99)	–	–	–	–

Among patients with SAD, 1 study involving 7 machine learning models was included. XGBoost achieved the highest AUC (0.74, 95% CI: 0.71–0.78), whereas RF yielded the lowest AUC (0.53, 95% CI: 0.48–0.58). The pooled AUC was 0.67 (95% CI: 0.63–0.72). See Table 4. The pooled sensitivity was 0.97 (95% CI: 0.94–0.98), and the pooled specificity was 0.11 (95% CI: 0.06–0.17). See Supplementary S4 Fig in S1 File.

For patients with SAE, 3 studies involving 10 machine learning models were included. LR was the most frequently evaluated model (n = 3), with an AUC of 0.81 (95% CI: 0.78–0.85). AdaBoost, NNET, XGBoost, CatBoost, RF, and BT each had an AUC of 0.83 (n = 1 each). The pooled AUC was 0.80 (95% CI: 0.79–0.82). See Table 4. The pooled sensitivity was 0.80 (95% CI: 0.77–0.82), and the pooled specificity was 0.73 (95% CI: 0.70–0.75). See Supplementary S5 Fig in S1 File.

For patients with SAKI, 4 studies involving 6 machine learning models were included. LR was the most frequently evaluated model (n = 2), with an AUC of 0.76 (95% CI: 0.74–0.78). Among all machine learning models, RSF achieved the highest AUC (0.85, 95% CI: 0.83–0.86), whereas CART yielded the lowest AUC (0.66, 95% CI: 0.62–0.71). The pooled AUC was 0.79 (95% CI: 0.77–0.81). See Table 4. The pooled sensitivity was 0.79 (95% CI: 0.75–0.83), and the pooled specificity was 0.64 (95% CI: 0.60–0.68). See Supplementary S6 Fig in S1 File.

For patients with SIC, 4 studies involving 10 machine learning models were included. LR was the most frequently evaluated model (n = 3), with an AUC of 0.82 (95% CI: 0.80–0.84). Among all machine learning models, XGBoost (n = 2) achieved the highest AUC (0.88, 95% CI: 0.84–0.91), whereas KNN yielded the lowest AUC (0.62, 95% CI: 0.55–0.69). The pooled AUC was 0.80 (95% CI: 0.77–0.82). See Table 4. The pooled sensitivity was 0.63 (95% CI: 0.56–0.70), and the pooled specificity was 0.78 (95% CI: 0.73–0.83). See Supplementary S7 Fig in S1 File.

Among patients with SICM, 1 study involving 8 machine learning models was included. LR achieved the highest AUC (0.82, 95% CI: 0.74–0.89), whereas KNN yielded the lowest AUC (0.70, 95% CI: 0.61–0.78). The pooled AUC was 0.78 (95% CI: 0.75–0.81). See Table 4. The pooled sensitivity was 0.75 (95% CI: 0.67–0.81), and the pooled specificity was 0.67 (95% CI: 0.63–0.72). See Supplementary S8 Fig in S1 File.

Among patients with SIMI, only one LR model was included, with an AUC of 0.77 (95% CI: 0.76–0.78). See Table 4.

Among patients with septic shock, only one LR model was included, with an AUC of 0.95 (95% CI: 0.85–0.99). See Table 4.

#### 3.4.4 The machine learning model predicted long-term mortality with an AUC value based on the validation set.

Among patients with SAE, only one LR model was included, with an AUC of 0.86 (95% CI: 0.83–0.89). See Table 4.

Among patients with SCRS, 1 study involving 6 machine learning models was included. RF achieved the highest AUC (0.83, 95% CI: 0.78–0.87), whereas SVM yielded the lowest AUC (0.68, 95% CI: 0.62–0.73). The pooled AUC was 0.76 (95% CI: 0.72–0.81). See Table 4.

#### 3.4.5 Sensitivity analysis.

Given the limited number of studies on sepsis-related complication outcomes, the sensitivity analyses in this study focused on the primary outcome of machine learning–based mortality prediction in patients with sepsis. The results showed that, in both the training and validation sets, excluding any single study had a negligible impact on the summary effect estimates across all model evaluation metrics. This finding confirms that no individual study substantially influenced overall predictive performance or heterogeneity, indicating that the pooled results of this meta-analysis are robust and reliable. See Supplementary S9 and S10 Figs in S1 File.

#### 3.4.6 Publication bias.

In the training set, a funnel plot was constructed from the model AUCs of the included studies to assess publication bias. The funnel plot was asymmetric, as shown in S11 Fig in S1 File. Egger’s test yielded t = 2.13, p = 0.039, confirming publication bias. See Supplementary S12 Fig in S1 File. A trim-and-fill analysis was then performed, estimating and imputing 17 missing studies. After trim-and-fill, the pooled AUC under the random-effects model was 0.78 (95% CI: 0.76–0.79), compared with the original pooled AUC of 0.82 (95% CI: 0.81–0.83), representing a decrease of approximately 4%. This suggests that the original pooled estimate might have been mildly overestimated due to publication bias. Given the relatively large number of imputed studies (36% of the original studies), the possibility of overcorrection should be considered for the trim-and-fill estimate, and the results should be interpreted with caution. See Supplementary S13 Fig in S1 File.

In the validation set, the funnel plot results indicated that the studies were not symmetrically distributed on either side, as shown in S14 Fig in S1 File. Egger’s test showed t = −0.79, p = 0.466, confirming the absence of publication bias. See Supplementary S15 Fig in S1 File.

## 4. Discussion

### 4.1 Principal findings

#### 4.1.1 Short-term mortality.

The overall performance of machine learning models for predicting short-term mortality in patients with sepsis was moderate, with a pooled AUC of 0.82 in the training set (46 studies, 15 model types) and 0.79 in the validation set (50 studies, 25 model types), representing a decrease of approximately 3.7% from the training set to the validation set, suggesting a certain degree of generalizability. At the model type level, LR was the most extensively evaluated model (n = 46 in the validation set), with a training-set AUC of 0.84 and a validation-set AUC of 0.81, demonstrating stable performance and strong interpretability, making it a reliable baseline choice for clinical application. RF and XGBoost performed comparably to logistic regression, with AUCs consistently ranging between 0.81 and 0.83 in both the training and validation sets. LightGBM achieved the highest AUC in the training set (0.92), and DL-CMT achieved the highest AUC in the validation set (0.94), but both were based on only one study, and their superiority requires confirmation through external validation. Overall, ensemble learning methods such as RF and XGBoost did not show a substantial advantage over traditional logistic regression in this population, although the outstanding performance of advanced algorithms in individual studies warrants attention.

SAE was among the complications with the most robust evidence base. LR (n = 3) yielded a pooled AUC of 0.81, demonstrating stable performance and serving as a reliable model for short-term mortality prediction in this complication. SIC performed comparably to SAE, with a pooled AUC of 0.80 in the validation set (4 studies, 10 model types). Notably, its training-set AUC was only 0.73, whereas the validation-set AUC surpassed that of the training set, suggesting that the studies or models in the validation set provided more consistent predictive signals. XGBoost (n = 2) achieved the best performance in this complication (AUC = 0.88), outperforming RF (AUC = 0.84), indicating a clear advantage of ensemble learning methods in this area. SAKI showed moderate and stable performance in both the training set (AUC = 0.78) and the validation set (AUC = 0.79), with a relatively sufficient evidence base (4 studies in the validation set). LR and SVM both achieved an AUC of 0.79 in the training set, while RSF achieved the highest AUC in the validation set (0.85), albeit based on only one study. SICM exhibited a notable discrepancy in performance between the training and validation sets, with a training-set AUC of 0.84 (1 study, 8 models) decreasing to 0.78 in the validation set (1 study, 8 models). RF performed exceptionally well in the training set (AUC = 0.98), but the AUCs of all models in the validation set were concentrated between 0.70 and 0.82, suggesting that the outstanding performance in the training set might stem from the specific data characteristics of a single study and lacks external generalizability.

Septic shock demonstrated excellent predictive performance (AUC = 0.95). SIMI had an AUC of 0.77, and SAD had a pooled AUC of only 0.67. All three complications were based on a single study, indicating an extremely weak evidence base. Although septic shock showed promising results, any conclusions require substantial external validation. The AUC for SAD being below 0.70 suggests that conventional machine learning models have limited predictive ability for this complication, and it may be necessary to incorporate delirium-specific risk factors, such as sedative use and delirium screening scales.

#### 4.1.2 Long-term mortality.

Research on long-term mortality prediction is extremely limited. In the training set, only SAE reported relevant data (1 LR study, AUC 0.88); in the validation set, SAE also reported only 1 LR study (AUC 0.86). For SCRS, 1 study involving 6 models was identified, with a pooled AUC of 0.76 (RF highest at 0.83, SVM lowest at 0.68). Overall, the evidence is derived from single studies, precluding robust conclusions, and the clinical value of machine learning models for long-term prognosis assessment in patients with sepsis remains to be further validated.

### 4.2 Comparison of performance on the training set and validation set

This study found that, for short-term mortality outcomes in sepsis, the summary AUC across all models on the training set was 0.82 (95% CI: 0.81–0.83), whereas it decreased to 0.79 (95% CI: 0.78–0.80) on the validation set, representing a reduction of approximately 3.7%. This performance decay was within the expected range and may be attributable to heterogeneity arising from differences in patients’ underlying diseases, illness severity, the distribution of input variables in the models, the temporal definition of mortality, and data sources. It may also be related to potential overfitting in internally validated models. Specifically, LR decreased from an AUC of 0.84 on the training set to 0.81 on the validation set, whereas RF and XGBoost remained relatively stable across both datasets (AUC 0.81–0.83). Of note, several models evaluated only in the validation set (e.g., DL-CMT, DNN) showed higher point estimates (AUC 0.84–0.94), but all were based on single studies, and their generalizability requires further confirmation. Nevertheless, the validation set results are more reflective of real-world clinical application scenarios; therefore, we recommend using the summary results from the validation set as the primary reference for efficacy evaluation and emphasize the importance of reporting external validation results in future primary studies, while training set results may serve only as internal references during model development.

### 4.3 Prediction differences between subgroups of different complications

In the validation set, predictive performance across complication subgroups showed a clear gradient: SAE (AUC = 0.80) and SIC (AUC = 0.80) performed best, followed by overall sepsis (0.79), SAKI (0.79), and SICM (0.78), whereas SAD had the lowest predictive efficacy (AUC = 0.67). This disparity may stem from multiple factors. First, the favorable performance of machine learning models in SAE and SIC may be partly attributed to the inclusion of predictive factors that are more readily identified and extracted by the models. In the case of SAE, three independent studies consistently identified BUN, lactate, and the Charlson Comorbidity Index (CCI) as key predictors that survived variable selection via LASSO regression or RFE among a large pool of candidate variables [36,37,54]. Mechanistically, BUN reflects the burden of blood–brain barrier toxin exposure [94]; elevated lactate may indicate neutrophil migration between the skull and the meninges, thereby exacerbating neuroinflammatory responses in severe brain injury [95]; and CCI reflects the patient’s overall physiological function [96]—all of which have been shown to reliably correlate with prognosis. Therefore, these three variables should be considered core components when constructing predictive models for SAE. Across the three included studies on SIC, the SOFA score and lactate were identified as core predictive factors [62,71,97]. The SOFA score provides a comprehensive assessment of multi-organ dysfunction [98], while lactate reflects tissue hypoperfusion and microcirculatory disturbances [99], which interact with coagulation dysfunction in a reciprocal cause-and-effect manner. In addition, RDW demonstrated a high importance ranking in the SHAP analysis of Wu et al. and exhibited a relatively large effect size in Yang et al. (OR = 1.24), suggesting that it is an emerging predictive indicator worthy of attention in the field of SIC.

### 4.4 Comparison of different model types

Across machine learning models, we identified several key findings. First, LR was the most frequently evaluated model (n = 27 in the training set, n = 46 in the validation set), with a summary AUC of 0.81 (95% CI: 0.79–0.83) in the validation set, demonstrating robust performance and strong interpretability, making it a reasonable choice for assessing clinical prognostic modeling. Second, among ensemble learning methods, such as XGBoost, RF, and LightGBM, performance was superior to or comparable with that of LR, with particularly notable advantages observed in SIC (XGBoost AUC = 0.88) in the validation set and SICM (RF AUC = 0.98) in the training set, consistent with the theoretical strength of ensemble learning in capturing nonlinear relationships and feature interactions. Third, deep learning methods, such as DL-CMT, achieved the highest AUC (0.94, 95% CI: 0.91–0.97) in the validation set for sepsis patients; however, this result was based on only one study and is insufficient to support the conclusion that deep learning is universally superior to traditional machine learning. Fourth, some models exhibited suboptimal performance, such as KNN, DT, and CART, which may be attributed to their sensitivity to data noise or susceptibility to overfitting, limiting their applicability in the highly heterogeneous data environment of the ICU.

Finally, it is worth noting that LightGBM performed best on the training set for sepsis patients (AUC = 0.92), whereas DL-CMT (0.94) and XGBoost (0.88) led on the validation set. This pattern, in which the “best model” varies across datasets, may suggest that optimal models derived from single-center, small-sample studies lack generalizability and that external generalizability is the key criterion for successful clinical translation.

### 4.5 Clinical significance and application prospects

Although the summary AUC of machine learning models for predicting short-term mortality in patients with sepsis is approximately 0.80, which has not yet reached the threshold for an ideal diagnostic test, this effect size still has clinically meaningful value given the highly complex pathophysiology of sepsis. As early prognostic risk stratification tools, machine learning models can generate dynamic risk scores at multiple time points after patient admission, such as at ICU admission and at 24 and 48 hours post-admission. These scores can then be integrated with clinicians’ judgment to aid and optimize disease monitoring and early intervention. Furthermore, the balanced performance in sensitivity (approximately 0.75–0.80) and specificity (approximately 0.74–0.80) observed in this study suggests that such models can be used both for “ruling out” low-risk patients and for “ruling in” high-risk patients, depending on the specific clinical application context. Future research should focus particularly on the SAE and SIC domains, while there is an urgent need to fill the data gaps in SAD and SIMI.

### 4.6 Limitations

This study has several important limitations. First, our findings showed substantial unexplained heterogeneity. Although we used a random-effects model to address this, subgroup analyses could not fully account for the sources of heterogeneity. There may be considerable differences among the included studies in terms of patient populations, including variations in clinical settings such as the emergency department, intensive care unit, or general ward, geographic regions, and baseline case-mix severity. In addition, outcome definitions varied, with mortality assessed at different time points such as in-hospital, 28-day, 30-day, and 90-day mortality. Furthermore, the time windows for mortality prediction were inconsistent across models, which directly affected the reported bias. Finally, there were differences in model development methods, including different algorithms such as logistic regression, random forest, gradient boosting, and deep learning, as well as feature selection strategies and validation methods. These differences in patient populations, outcome definitions, prediction horizons, and model development methods may be the main sources of the observed heterogeneity and should be taken into account when interpreting the pooled estimates. Second, nearly half of the included studies were conducted at single centers, resulting in limited sample sizes available for evaluation, which may have led to selection bias, overfitting, and insufficient generalizability of the findings. Third, only a few studies performed true external validation on completely independent sample sets, which may have overestimated the actual performance of the models in new populations and limited our ability to comprehensively assess model generalizability. Fourth, although the trim-and-fill method was applied to correct for publication bias in the training set, this method is based on the assumption of symmetry, and when the number of missing studies is substantial (accounting for 36% of the original studies), the robustness of the corrected results should be interpreted with caution. Finally, our study was unable to assess the calibration performance or clinical utility of these models. Most primary studies only reported discriminative ability (AUC) without presenting calibration curves or calibration slopes, which prevented us from evaluating the agreement between predicted probabilities and observed outcomes.

## 5. Conclusions

This meta-analysis shows that machine learning models have moderate discriminative ability for predicting short-term mortality in patients with sepsis. LR, the most extensively evaluated model, shows stable performance. RF and XGBoost perform comparably to logistic regression in the overall sepsis population, though they show promising advantages in specific complications. Among the complication subgroups, SAE, SIC, and SAKI have relatively robust evidence bases and demonstrate favorable predictive performance, whereas septic shock, SICM, SAD, and SIMI are supported by only single-study evidence and exhibit limited predictive utility. Regarding long-term mortality, evidence is extremely scarce, with all estimates derived from single studies, precluding any robust conclusions.

Therefore, machine learning models hold promise as early prognostic risk assessment tools for the management of sepsis and its related complications, yet their clinical generalizability requires further external validation. Future research should prioritize well-designed, multicenter prospective studies with rigorous external validation, particularly for under-investigated complications, to facilitate the eventual integration of these models into routine clinical practice.

## Supporting information

S1 File S1 Table. PRISMA 2020 checklist. S2 Table. Search strategy. S3 Table. Methodological quality assessment. S1 Fig. Sensitivity and specificity for short-term mortality in patients with sepsis in the training set. S2 Fig. Sensitivity and specificity for short-term mortality in patients with SAKI in the training set. S3 Fig. Sensitivity and specificity for short-term mortality in patients with SICM in the training set. S4 Fig. Sensitivity and specificity for short-term mortality in patients with SAD in the validation set. S5 Fig. Sensitivity and specificity for short-term mortality in patients with SAE in the validation set. S6 Fig. Sensitivity and specificity for short-term mortality in patients with SAKI in the validation set. S7 Fig. Sensitivity and specificity for short-term mortality in patients with SIC in the validation set. S8 Fig. Sensitivity and specificity for short-term mortality in patients with SICM in the validation set. S9 Fig. Sensitivity analysis for short-term mortality in sepsis in the training set. S10 Fig. Sensitivity analysis for short-term mortality in sepsis in the validation set. S11 Fig. Funnel plot for short-term mortality in sepsis in the training set. S12 Fig. Egger’s test for short-term mortality in sepsis in the training set. S13 Fig. Trim-and-fill method for short-term mortality in sepsis in the training set. S14 Fig. Funnel plot for short-term mortality in sepsis in the validation set. S15 Fig. Egger’s test for short-term mortality in sepsis in the validation set.
(DOCX)

S2 File The supplementary material containing the raw data for all outcomes evaluated in our study has been named “Raw Outcome Data”. This file provides the study-level extracted data—including AUC, sensitivity, and specificity—for all included studies and outcomes, which served as the basis for our meta-analyses. (XLSX)
