# Supplementary material for: Machine learning for predicting mortality in patients with sepsis and its associated complications: A systematic review and meta-analysis
Source: PLoS One. 2026 Nov 2;21(11):e0362057. doi: 10.1371/journal.pone.0362057 (PMC13726949; doi:10.1371/journal.pone.0362057)
Supplement: Supplementary file 1 [file pone.0362057.s001.docx]

**Table S1: PRISMA 2020 checklist.**

| **Section and Topic** | **Item #** | **Checklist item** | **Location where item is reported** |
| --- | --- | --- | --- |
| **TITLE** | | |  |
| Title | 1 | Identify the report as a systematic review. | 1 |
| **ABSTRACT** | | |  |
| Abstract | 2 | See the PRISMA 2020 for Abstracts checklist. | 2 |
| **INTRODUCTION** | | |  |
| Rationale | 3 | Describe the rationale for the review in the context of existing knowledge. | 3 |
| Objectives | 4 | Provide an explicit statement of the objective(s) or question(s) the review addresses. | 3 |
| **METHODS** | | |  |
| Eligibility criteria | 5 | Specify the inclusion and exclusion criteria for the review and how studies were grouped for the syntheses. | 4 |
| Information sources | 6 | Specify all databases, registers, websites, organisations, reference lists and other sources searched or consulted to identify studies. Specify the date when each source was last searched or consulted. | 4 |
| Search strategy | 7 | Present the full search strategies for all databases, registers and websites, including any filters and limits used. | 4 |
| Selection process | 8 | Specify the methods used to decide whether a study met the inclusion criteria of the review, including how many reviewers screened each record and each report retrieved, whether they worked independently, and if applicable, details of automation tools used in the process. | 5 |
| Data collection process | 9 | Specify the methods used to collect data from reports, including how many reviewers collected data from each report, whether they worked independently, any processes for obtaining or confirming data from study investigators, and if applicable, details of automation tools used in the process. | 5 |
| Data items | 10a | List and define all outcomes for which data were sought. Specify whether all results that were compatible with each outcome domain in each study were sought (e.g. for all measures, time points, analyses), and if not, the methods used to decide which results to collect. | 5 |
|  | 10b | List and define all other variables for which data were sought (e.g. participant and intervention characteristics, funding sources). Describe any assumptions made about any missing or unclear information. | 5 |
| Study risk of bias assessment | 11 | Specify the methods used to assess risk of bias in the included studies, including details of the tool(s) used, how many reviewers assessed each study and whether they worked independently, and if applicable, details of automation tools used in the process. | 6 |
| Effect measures | 12 | Specify for each outcome the effect measure(s) (e.g. risk ratio, mean difference) used in the synthesis or presentation of results. | 6 |
| Synthesis methods | 13a | Describe the processes used to decide which studies were eligible for each synthesis (e.g. tabulating the study intervention characteristics and comparing against the planned groups for each synthesis (item #5)). | 6 |
|  | 13b | Describe any methods required to prepare the data for presentation or synthesis, such as handling of missing summary statistics, or data conversions. | 6 |
|  | 13c | Describe any methods used to tabulate or visually display results of individual studies and syntheses. | 6 |
|  | 13d | Describe any methods used to synthesize results and provide a rationale for the choice(s). If meta-analysis was performed, describe the model(s), method(s) to identify the presence and extent of statistical heterogeneity, and software package(s) used. | 6 |
|  | 13e | Describe any methods used to explore possible causes of heterogeneity among study results (e.g. subgroup analysis, meta-regression). | 6 |
|  | 13f | Describe any sensitivity analyses conducted to assess robustness of the synthesized results. | 6 |
| Reporting bias assessment | 14 | Describe any methods used to assess risk of bias due to missing results in a synthesis (arising from reporting biases). | 6 |
| Certainty assessment | 15 | Describe any methods used to assess certainty (or confidence) in the body of evidence for an outcome. | 6 |
| **RESULTS** | | |  |
| Study selection | 16a | Describe the results of the search and selection process, from the number of records identified in the search to the number of studies included in the review, ideally using a flow diagram. | 6 |
|  | 16b | Cite studies that might appear to meet the inclusion criteria, but which were excluded, and explain why they were excluded. | 6 |
| Study characteristics | 17 | Cite each included study and present its characteristics. | 7 |
| Risk of bias in studies | 18 | Present assessments of risk of bias for each included study. | 8 |
| Results of individual studies | 19 | For all outcomes, present, for each study: (a) summary statistics for each group (where appropriate) and (b) an effect estimate and its precision (e.g. confidence/credible interval), ideally using structured tables or plots. | 8 |
| Results of syntheses | 20a | For each synthesis, briefly summarise the characteristics and risk of bias among contributing studies. | 8 |
|  | 20b | Present results of all statistical syntheses conducted. If meta-analysis was done, present for each the summary estimate and its precision (e.g. confidence/credible interval) and measures of statistical heterogeneity. If comparing groups, describe the direction of the effect. | 8-10 |
|  | 20c | Present results of all investigations of possible causes of heterogeneity among study results. | 8-10 |
|  | 20d | Present results of all sensitivity analyses conducted to assess the robustness of the synthesized results. | 8-10 |
| Reporting biases | 21 | Present assessments of risk of bias due to missing results (arising from reporting biases) for each synthesis assessed. | 11 |
| Certainty of evidence | 22 | Present assessments of certainty (or confidence) in the body of evidence for each outcome assessed. | 11 |
| **DISCUSSION** | | |  |
| Discussion | 23a | Provide a general interpretation of the results in the context of other evidence. | 12-14 |
|  | 23b | Discuss any limitations of the evidence included in the review. | 12-14 |
|  | 23c | Discuss any limitations of the review processes used. | 15 |
|  | 23d | Discuss implications of the results for practice, policy, and future research. | 15 |
| **OTHER INFORMATION** | | |  |
| Registration and protocol | 24a | Provide registration information for the review, including register name and registration number, or state that the review was not registered. | 4 |
|  | 24b | Indicate where the review protocol can be accessed, or state that a protocol was not prepared. | 4 |
|  | 24c | Describe and explain any amendments to information provided at registration or in the protocol. | 4 |
| Support | 25 | Describe sources of financial or non-financial support for the review, and the role of the funders or sponsors in the review. | 16 |
| Competing interests | 26 | Declare any competing interests of review authors. | 16 |
| Availability of data, code and other materials | 27 | Report which of the following are publicly available and where they can be found: template data collection forms; data extracted from included studies; data used for all analyses; analytic code; any other materials used in the review. | 17 |

*From:* Page MJ, McKenzie JE, Bossuyt PM, Boutron I, Hoffmann TC, Mulrow CD, et al. The PRISMA 2020 statement: an updated guideline for reporting systematic reviews. BMJ 2021;372:n71. doi: 10.1136/bmj.n71

For more information, visit: <http://www.prisma-statement.org/>

**Table S2: Search strategy.**

**Cochrane**

ID	Search	Hits

#1	MeSH descriptor: [Machine Learning] explode all trees	1403

#2	(machine learning OR transfer learning OR deep learning OR ensemble learning OR artificial intelligence OR prediction model OR random forest OR artificial neural network OR support vector machine OR gradient boosting machine OR nomogram OR xgboost OR decision tree OR risk prediction OR machine intelligence OR bayesian OR naïve bayes OR decision support OR adaboost OR neural network OR gradient boosting OR prediction model OR algorithm OR supervised learning OR ensemble method):ti,ab,kw	53447

#3	#1 OR #2	53465

#4	MeSH descriptor: [Sepsis] explode all trees	6825

#5	(sepsis OR bloodstream infection* OR pyemia* OR pyohemia* OR pyaemia OR pyaemias OR septicemia OR septicemias OR blood poisoning* OR septic disease OR septic):ti,ab,kw OR (septic shock OR sepsis associated encephalopathy OR sepsis associated acute kidney injury OR sepsis induced coagulopathy OR sepsis associated delirium OR sepsis induced cardiorespiratory syndrome OR sepsis induced cardiomyopathy OR sepsis induced myocardial injury):ti,ab,kw	21716

#6	#4 OR #5	23552

#7	(mortality OR mortalities OR case fatality rate* OR cfr case fatality rate OR mors OR death):ti,ab,kw	194517

#8	(cohort OR follow up OR longitudinal OR prospective OR retrospective OR observational OR prognostic)	718117

#9	#3 AND #6 AND #7 AND #8	260

**Pubmed**

| Search number | Search Details | Results |
| --- | --- | --- |
| 5 | (((cohort OR follow up OR longitudinal OR prospective OR retrospective OR observational OR prognostic) AND (mortality[Title/Abstract] OR mortalities[Title/Abstract] OR case fatality rate*[Title/Abstract] OR cfr case fatality rate[Title/Abstract] OR mors[Title/Abstract] OR death[Title/Abstract])) AND (((sepsis[MeSH Terms]) OR (sepsis[Title/Abstract] OR bloodstream infection*[Title/Abstract] OR pyemia*[Title/Abstract] OR pyohemia*[Title/Abstract] OR pyaemia[Title/Abstract] OR pyaemias[Title/Abstract] OR septicemia[Title/Abstract] OR septicemias[Title/Abstract] OR blood poisoning*[Title/Abstract] OR septic disease[Title/Abstract] OR septic[Title/Abstract])) OR (septic shock[Title/Abstract] OR sepsis associated encephalopathy[Title/Abstract] OR sepsis associated acute kidney injury[Title/Abstract] OR sepsis induced coagulopathy[Title/Abstract] OR sepsis associated delirium[Title/Abstract] OR sepsis induced cardiorespiratory syndrome[Title/Abstract] OR sepsis induced cardiomyopathy[Title/Abstract] OR sepsis induced myocardial injury[Title/Abstract]))) AND ((machine learning[MeSH Terms]) OR (machine learning[Title/Abstract] OR transfer learning[Title/Abstract] OR deep learning[Title/Abstract] OR ensemble learning[Title/Abstract] OR artificial intelligence[Title/Abstract] OR prediction model[Title/Abstract] OR random forest[Title/Abstract] OR artificial neural network[Title/Abstract] OR support vector machine[Title/Abstract] OR gradient boosting machine[Title/Abstract] OR nomogram[Title/Abstract] OR xgboost[Title/Abstract] OR decision tree[Title/Abstract] OR risk prediction[Title/Abstract] OR machine intelligence[Title/Abstract] OR bayesian[Title/Abstract] OR naïve bayes[Title/Abstract] OR decision support[Title/Abstract] OR adaboost[Title/Abstract] OR neural network[Title/Abstract] OR gradient boosting[Title/Abstract] OR prediction model[Title/Abstract] OR algorithm[Title/Abstract] OR supervised learning[Title/Abstract] OR ensemble method[Title/Abstract])) | 1,838 |
| 4 | cohort OR follow up OR longitudinal OR prospective OR retrospective OR observational OR prognostic | 5,573,915 |
| 3 | mortality[Title/Abstract] OR mortalities[Title/Abstract] OR case fatality rate*[Title/Abstract] OR cfr case fatality rate[Title/Abstract] OR mors[Title/Abstract] OR death[Title/Abstract] | 2,087,081 |
| 2 | ((sepsis[MeSH Terms]) OR (sepsis[Title/Abstract] OR bloodstream infection*[Title/Abstract] OR pyemia*[Title/Abstract] OR pyohemia*[Title/Abstract] OR pyaemia[Title/Abstract] OR pyaemias[Title/Abstract] OR septicemia[Title/Abstract] OR septicemias[Title/Abstract] OR blood poisoning*[Title/Abstract] OR septic disease[Title/Abstract] OR septic[Title/Abstract])) OR (septic shock[Title/Abstract] OR sepsis associated encephalopathy[Title/Abstract] OR sepsis associated acute kidney injury[Title/Abstract] OR sepsis induced coagulopathy[Title/Abstract] OR sepsis associated delirium[Title/Abstract] OR sepsis induced cardiorespiratory syndrome[Title/Abstract] OR sepsis induced cardiomyopathy[Title/Abstract] OR sepsis induced myocardial injury[Title/Abstract]) | 295,447 |
| 1 | (machine learning[MeSH Terms]) OR (machine learning[Title/Abstract] OR transfer learning[Title/Abstract] OR deep learning[Title/Abstract] OR ensemble learning[Title/Abstract] OR artificial intelligence[Title/Abstract] OR prediction model[Title/Abstract] OR random forest[Title/Abstract] OR artificial neural network[Title/Abstract] OR support vector machine[Title/Abstract] OR gradient boosting machine[Title/Abstract] OR nomogram[Title/Abstract] OR xgboost[Title/Abstract] OR decision tree[Title/Abstract] OR risk prediction[Title/Abstract] OR machine intelligence[Title/Abstract] OR bayesian[Title/Abstract] OR naïve bayes[Title/Abstract] OR decision support[Title/Abstract] OR adaboost[Title/Abstract] OR neural network[Title/Abstract] OR gradient boosting[Title/Abstract] OR prediction model[Title/Abstract] OR algorithm[Title/Abstract] OR supervised learning[Title/Abstract] OR ensemble method[Title/Abstract]) | 927,701 |

**Embase**

| No. | Query | Results |
| --- | --- | --- |
| #5 | #1 AND #2 AND #3 AND #4 | 3,173 |
| #4 | (cohort OR follow) AND up OR longitudinal OR prospective OR retrospective OR observational OR prognostic | 7,530,624 |
| #3 | mortality:ab,ti OR mortalities:ab,ti OR 'case fatality rate*':ab,ti OR 'cfr case fatality rate':ab,ti OR mors:ab,ti OR death:ab,ti | 3,096,771 |
| #2 | 'sepsis'/exp OR sepsis:ab,ti OR 'bloodstream infection*':ab,ti OR pyemia*:ab,ti OR pyohemia*:ab,ti OR pyaemia:ab,ti OR pyaemias:ab,ti OR septicemia:ab,ti OR septicemias:ab,ti OR 'blood poisoning*':ab,ti OR 'septic disease':ab,ti OR septic:ab,ti OR 'septic shock':ab,ti OR 'sepsis associated encephalopathy':ab,ti OR 'sepsis associated acute kidney injury':ab,ti OR 'sepsis induced coagulopathy':ab,ti OR 'sepsis associated delirium':ab,ti OR 'sepsis induced cardiorespiratory syndrome':ab,ti OR 'sepsis induced cardiomyopathy':ab,ti OR 'sepsis induced myocardial injury':ab,ti | 527,793 |
| #1 | 'machine learning'/exp OR 'machine learning':ab,ti OR 'transfer learning':ab,ti OR 'deep learning':ab,ti OR 'ensemble learning':ab,ti OR 'artificial intelligence':ab,ti OR 'random forest':ab,ti OR 'artificial neural network':ab,ti OR 'support vector machine':ab,ti OR 'gradient boosting machine':ab,ti OR nomogram:ab,ti OR xgboost:ab,ti OR 'decision tree':ab,ti OR 'risk prediction':ab,ti OR 'machine intelligence':ab,ti OR bayesian:ab,ti OR 'naïve bayes':ab,ti OR 'decision support':ab,ti OR adaboost:ab,ti OR 'neural network':ab,ti OR 'gradient boosting':ab,ti OR 'prediction model':ab,ti OR algorithm:ab,ti OR 'supervised learning':ab,ti OR 'ensemble method':ab,ti | 1,419,107 |

**Web of Science**

machine learning OR transfer learning OR deep learning OR ensemble learning OR artificial intelligence OR prediction model OR random forest OR artificial neural network OR support vector machine OR gradient boosting machine OR nomogram OR xgboost OR decision tree OR risk prediction OR machine intelligence OR bayesian OR naïve bayes OR decision support OR adaboost OR neural network OR gradient boosting OR prediction model OR algorithm OR supervised learning OR ensemble method (Topic) and sepsis OR bloodstream infection* OR pyemia* OR pyohemia* OR pyaemic OR pyaemia OR septicemia OR septicemias OR blood poisoning* OR septic disease OR septic OR septic shock OR sepsis associated encephalopathy OR sepsis associated acute kidney injury OR sepsis induced coagulopathy OR sepsis associated delirium OR sepsis induced cardiorespiratory syndrome OR sepsis induced cardiomyopathy OR sepsis induced myocardial injury (Topic) and mortality OR mortalities OR case fatality rate* OR cfr case fatality rate OR mors OR death (Topic) and cohort OR follow up OR longitudinal OR prospective OR retrospective OR observational OR prognostic (All Fields)

Results: 4279

**Table S3: Methodological quality assessment**

| **Author (year)** | **PROBAST** | | | | **Applicability** | | | **Overall** | |
| --- | --- | --- | --- | --- | --- | --- | --- | --- | --- |
|  | **Participants** | **Predictors** | **Outcome** | **Analysis** | **Participants** | **Predictors** | **Outcome** | **PROBAST** | **Applicability** |
| AnShuoyan 2025 | Low | Low | Low | High | Low | Low | Low | High | Low |
| ChenDaonan 2024 | Low | Low | Low | High | Low | High | Low | High | High |
| ChengChiyung 2022 | Low | Low | Low | High | Low | Low | Low | High | Low |
| ChenLinmu 2026 | Low | Low | Low | Unclear | Low | Low | Low | Unclear | Low |
| ChenXianyuan 2026 | Low | Low | Low | Unclear | Low | Low | Low | Unclear | Low |
| DrăgoescuAlicenicoleta 2026 | Low | Low | Low | Unclear | Low | Low | Low | Unclear | Low |
| GanZiyan 2026 | Low | Low | Low | High | Low | Low | Low | High | Low |
| GaoQiang 2025 | Low | Low | Low | High | Low | Low | Low | High | Low |
| GuoQirui 2024 | Low | Low | Low | High | Low | Low | Low | High | Low |
| HanKun 2025 | Low | Low | Low | Unclear | Low | Low | Low | Unclear | Low |
| HoangQuangvan 2025 | Low | Low | Low | Unclear | Low | Low | Low | Unclear | Low |
| HongChengying 2025 | Low | Low | Low | High | Low | Low | Low | High | Low |
| HuangYuwei 2025 | Low | Low | Low | High | Low | High | Low | High | High |
| HuChang 2022 | Low | Low | Low | High | Low | Low | Low | High | Low |
| HuXuemei 2025 | Low | Low | Low | High | Low | Low | Low | High | Low |
| JinGuangyong 2024 | Low | Low | Low | High | Low | Low | Low | High | Low |
| JinJun 2026 | Low | Low | Low | Unclear | Low | Low | Low | Unclear | Low |
| LiCaifeng 2024 | Low | Low | Low | High | Low | Low | Low | High | Low |
| LiHaitao 2023 | Low | Low | Low | Unclear | Low | Low | Low | Unclear | Low |
| LiQiujing 2023 | Low | Low | Low | Unclear | Low | Low | Low | Unclear | Low |
| LiuTing 2026 | Low | Low | Low | Unclear | Low | Low | Low | Unclear | Low |
| LiuXuhui 2024 | Low | Low | Low | High | Low | Low | Low | High | Low |
| LiuYiguo 2022 | Low | Low | Low | Unclear | Low | Low | Low | Unclear | Low |
| LiXiang 2026 | Low | Low | Low | High | Low | Low | Low | High | Low |
| LiYongkai 2023 | Low | Low | Low | High | Low | High | Low | High | High |
| LiYujie 2026 | Low | Low | Low | High | Low | Low | Low | High | Low |
| LuoXiang 2025 | Low | Low | Low | Unclear | Low | Low | Low | Unclear | Low |
| LuoXiaoqin 2022 | Low | Low | Low | Unclear | Low | Low | Low | Unclear | Low |
| LuoYali2025 | Low | Low | Low | Unclear | Low | Low | Low | Unclear | Low |
| MaChengyong 2023 | Low | Low | Low | Unclear | Low | Low | Low | Unclear | Low |
| MaXia 2025 | Low | Low | Low | Unclear | Low | Low | Low | Unclear | Low |
| OkoyeChukwuma 2025 | Low | Low | Low | High | Low | Low | Low | High | Low |
| PengLiwei 2022 | Low | Low | Low | Unclear | Low | Low | Low | Unclear | Low |
| PlutaMichałP 2024 | Low | Low | Low | Unclear | Low | Low | Low | Unclear | Low |
| QinHongmei 2025 | Low | Low | Low | Unclear | Low | Low | Low | Unclear | Low |
| ShenLi 2025 | Low | Low | Low | Unclear | Low | Low | Low | Unclear | Low |
| SheXiao 2025 | Low | Low | Low | Unclear | Low | Low | Low | Unclear | Low |
| SongJuhyun 2020 | Low | Low | Low | Unclear | Low | Low | Low | Unclear | Low |
| SuLongxiang 2021 | Low | Low | Low | Unclear | Low | Low | Low | Unclear | Low |
| SunYi 2025 | Low | Low | Low | Unclear | Low | Low | Low | Unclear | Low |
| TanRuimin 2025 | Low | Low | Low | Unclear | Low | Low | Low | Unclear | Low |
| UnalCetinEce 2025 | Low | Low | Low | Unclear | Low | Low | Low | Unclear | Low |
| WangQinxin 2025 | Low | Low | Low | Unclear | Low | Low | Low | Unclear | Low |
| WangXiaoqian 2024 | Low | Low | Low | Unclear | Low | Low | Low | Unclear | Low |
| WangYiping 2025 | Low | Low | Low | Unclear | Low | Low | Low | Unclear | Low |
| WeiJian 2024 | Low | Low | Low | Unclear | Low | Low | Low | Unclear | Low |
| WeiJuan 2025 | Low | Low | Low | Unclear | Low | Low | Low | Unclear | Low |
| WengLulu 2026 | Low | Low | Low | Unclear | Low | Low | Low | Unclear | Low |
| WuHongsheng 2024 | Low | Low | Low | Unclear | Low | Low | Low | Unclear | Low |
| WuJinmei 2026 | Low | Low | Low | Unclear | Low | Low | Low | Unclear | Low |
| XieYinjing 2023 | Low | Low | Low | Unclear | Low | Low | Low | Unclear | Low |
| XinQi 2026a | Low | Low | Low | Unclear | Low | Low | Low | Unclear | Low |
| XinQi 2026 | Low | Low | Low | Unclear | Low | Low | Low | Unclear | Low |
| XuXin 2026 | Low | Low | Low | Unclear | Low | Low | Low | Unclear | Low |
| XuYaya 2022 | Low | Low | Low | Unclear | Low | Low | Low | Unclear | Low |
| XuYifei 2026 | Low | Low | Low | Unclear | Low | Low | Low | Unclear | Low |
| XuZhikun 2026 | Low | Low | Low | Unclear | Low | Low | Low | Unclear | Low |
| YangJianyue 2025 | Low | Low | Low | Unclear | Low | Low | Low | Unclear | Low |
| YangHao2025 | Low | Low | Low | Unclear | Low | Low | Low | Unclear | Low |
| YangMeicheng 2024 | Low | Low | Low | Unclear | Low | Low | Low | Unclear | Low |
| YangYang 2022 | Low | Low | Low | Unclear | Low | Low | Low | Unclear | Low |
| YangYanjie 2025 | Low | Low | Low | Unclear | Low | Low | Low | Unclear | Low |
| YangYong 2022 | Low | Low | Low | Unclear | Low | Low | Low | Unclear | Low |
| YinJingjing 2026 | Low | Low | Low | Unclear | Low | Low | Low | Unclear | Low |
| YuXiaoyuan 2025 | Low | Low | Low | Unclear | Low | Low | Low | Unclear | Low |
| ZengQingbo 2021 | Low | Low | Low | Unclear | Low | Low | Low | Unclear | Low |
| ZengZhixuan 2021 | Low | Low | Low | Unclear | Low | Low | Low | Unclear | Low |
| ZhangDalong 2025 | Low | Low | Low | Unclear | Low | Low | Low | Unclear | Low |
| ZhangGuyu 2024 | Low | Low | Low | Unclear | Low | Low | Low | Unclear | Low |
| ZhangJing 2026 | Low | Low | Low | Unclear | Low | Low | Low | Unclear | Low |
| ZhouShu 2024 | Low | Low | Low | Unclear | Low | Low | Low | Unclear | Low |
| ZhouXuan 2025 | Low | Low | Low | Unclear | Low | Low | Low | Unclear | Low |

Figure S1 Sensitivity and specificity for short-term mortality in patients with sepsis in the training set.

Figure S2 Sensitivity and specificity for short-term mortality in patients with SAKI in the training set.

Figure S3 Sensitivity and specificity for short-term mortality in patients with SICM in the training set.

Figure S4 Sensitivity and specificity for short-term mortality in patients with SAD in the validation set.

Figure S5 Sensitivity and specificity for short-term mortality in patients with SAE in the validation set.

Figure S6 Sensitivity and specificity for short-term mortality in patients with SAKI in the validation set.

Figure S7 Sensitivity and specificity for short-term mortality in patients with SIC in the validation set.

Figure S8 Sensitivity and specificity for short-term mortality in patients with SICM in the validation set.

 Figure S9 Sensitivity analysis for short-term mortality in sepsis in the training set. Figure S10 Sensitivity analysis for short-term mortality in sepsis in the validation set.

Figure S11 Funnel plot for short‑term mortality in sepsis in the training set. Figure S12 Egger's test for short‑term mortality in sepsis in the training set.

Figure S13 Trim-and-fill method for short‑term mortality in sepsis in the training set. Figure S14 Funnel plot for short‑term mortality in sepsis in the validation set.

Figure S15 Egger's test for short‑term mortality in sepsis in the validation set.
